# Morpho-biochemical diversity and phytochemical profiling of *Rubus fruticosus* L. landraces

**DOI:** 10.1371/journal.pone.0350451

**Published:** 2026-07-02

**Authors:** Muhammad Owais, Imran Khan, Mohammad Nisar, Asghar Khan, Mohammad Ihsan, Nausheen Nazir, Farhana Ali, Muhammad Zahoor, Sumbal Khan, Shiou Yih Lee

**Affiliations:** 1 Department of Botany, University of Malakand, Chakdara, Khyber Pakhtunkhwa, Pakistan; 2 Department of Botany, Government Degree College Totakan, Malakand, Khyber Pakhtunkhwa, Pakistan; 3 Department of Biochemistry, University of Malakand, Chakdara, Khyber Pakhtunkhwa, Pakistan; 4 Atta-ur-Rahman School of Applied Biosciences, National University of Sciences and Technology, Islamabad, Pakistan; 5 Faculty of Health and Life Sciences, INTI International University, Nilai, Negeri Sembilan, Malaysia; University of Balochistan, PAKISTAN

## Abstract

The flora of Khyber Pakhtunkhwa, Pakistan, is severely threatened by illegal harvesting of non-timber forest resources, resulting in the genetic loss of native plant species, including *Rubus fruticosus* L., a genetically diverse shrub of considerable economic and medicinal importance. The present study was designed to assess the morphological diversity, seed protein profiles, and phytochemical composition of fifteen landraces of *R. fruticosus* collected from different ecological zones of the Malakand Division, Khyber Pakhtunkhwa, Pakistan. Morphological characteristics were evaluated using the International Board for Plant Genetic Resources (IBPGR) descriptors, incorporating both qualitative and quantitative traits in a randomized complete block design. Seed protein diversity was assessed through sodium dodecyl sulfate–polyacrylamide gel electrophoresis (SDS-PAGE), while phytochemical composition was quantified using high-performance liquid chromatography with ultraviolet detection (HPLC-UV). Morphological traits exhibited significant variability, with a mean coefficient of variation (CV) of 36.72%. The most variable traits were single fruit weight (CV = 73.16%), five-fruit weight (CV = 64.38%), number of branches per plant (CV = 33.33%), plant height (CV = 31.31%), and stalk length (CV = 30.11%). Principal component analysis (PCA) explained 89.71% of the total variation, with plant height, leaflet number, and fruit weight contributing predominantly to landrace differentiation. SDS-PAGE analysis of seed proteins revealed 21 polymorphic bands (molecular weight range: 14.4–97.4 kDa), with high diversity indices recorded for markers B-03, B-06, B-08, B-12, and B-21 (Shannon diversity index H′ = 1.10–1.39; CV = 35.36–93.54%). HPLC-UV analysis identified twelve phenolic compounds in selected genotypes, including quercetin (5.12–8.74 µg/mL), morin (3.45–6.89 µg/mL), and epigallocatechin gallate (2.88–7.23 µg/mL). The morphological, biochemical, and phytochemical diversity observed among *R. fruticosus* landraces highlights their potential for conservation programs and future breeding initiatives.

## Introduction

Wild edible fruits contribute substantially to human nutrition and food security, particularly in rural and indigenous communities. They constitute rich natural sources of essential vitamins, minerals, dietary fiber, and antioxidant phytochemicals—most notably flavonoids and anthocyanins—that mitigate oxidative damage and reduce the risk of chronic non-communicable diseases [1–3]. Beyond providing nutritional sustenance during periods of food scarcity, many wild fruit species are protein-rich, cost-effective, and serve as valuable dietary supplements for resource-limited populations [2]. In addition to their nutritional significance, these species also play an important role in biodiversity conservation and the sustainability of agroecosystems [3,4].

The blackberry, *Rubus fruticosus* L. (Rosaceae), is a wild fruit distributed across temperate regions of Pakistan, with documented occurrences in Mansehra [5], Chitral [6], and Kotli [7]. Locally, the species is referred to as “Karwara” [8], “Ach” [9], and “Akhara” [7]. Blackberries are valued for their flavor, nutritional composition, and wide applications in processed food products, including frozen desserts, jellies, and jams [10]. In addition to their culinary applications, blackberries are increasingly recognized for their abundance of bioactive compounds and purported biological activities, making them attractive candidates for functional food development [11,12].

*R. fruticosus* holds significant conservation and cultural importance. Its flowers provide a critical nectar source for pollinators, especially honeybees, supporting the production of light, aromatic honey [13]. Historically, the plant has been cultivated as a living barrier for boundary demarcation and crop protection [14]. These diverse ecological and cultural roles underscore the necessity of continued scientific investigation into this species [15–18].

The bioactive constituents of *R. fruticosus*—including minerals, vitamins, and phenolic compounds—underpin its classification as a functional food [11]. Traditional medicine systems have long employed this species for the treatment of respiratory ailments, coughs, and oral ulcers [19,20]. Pharmacological investigations have demonstrated antimicrobial, anti-allergic, diuretic, and anticoagulant activities [21]. The species is additionally rich in essential nutrients, including carotenoids, calcium, potassium, magnesium, vitamin C, and B-complex vitamins [15,16,22]. Its antioxidant and therapeutic properties are primarily attributed to bioactive compounds such as ellagic acid, anthocyanins, and related phytonutrients [23,24].

Despite the well-documented health benefits of blackberries, the preponderance of existing research has focused on commercially cultivated varieties. Native varieties—particularly those originating from Pakistan—remain substantially understudied with respect to their antioxidant potential and biochemical variability. This knowledge gap is significant, as concentrations of bioactive constituents in indigenous varieties can vary considerably depending on local environmental conditions and post-harvest processing methods [25,26]. Furthermore, limited information is available regarding the conservation status and biochemical diversity of indigenous landraces, which restricts their effective utilization in breeding and nutraceutical applications. Investigating the phytochemical profiles and antioxidant capacities of native *R. fruticosus* varieties is therefore essential to evaluate their potential as functional food ingredients and to identify superior candidates for medicinal product development [27].

Biochemical characterization encompassing the evaluation of phenolic constituents, anthocyanins, and antioxidant capacity is indispensable for quantifying the therapeutic potential of plant materials [28,29]. When integrated with multivariate analytical tools such as PCA, HCA, and PLS-DA, these approaches enable efficient characterization and discrimination of genotypes [30]. This integrated methodology has been successfully applied to berry species in Bosnia and Herzegovina [31], haskap, aronia, and goji berries in Spain [32], wild food plants in Malaysia [33,34], and *Ephedra alata* populations in Tunisia [35], demonstrated the broad applicability of phytochemical and functional analyses in germplasm evaluation.

Genetic variation is fundamental to crop improvement and facilitates the identification of superior cultivars [36,37]. Although molecular markers have proven valuable for characterizing genetic variation, morphological evaluation remains critical for assessing traits such as plant height, flowering time, seed protein and oil content, branching pattern, and resistance to biotic and abiotic stresses [38–40]. Furthermore, PCA applied to phylogenetic studies using genotyping-by-sequencing has revealed complex evolutionary relationships within the *Rubus* genus, highlighting remarkable morphological diversity and associated taxonomic challenges [41,42]. Identifying such diversity enables plant breeders to assess genetic relationships among genotypes and develop improved cultivars with enhanced yields and environmental adaptability [43,44].

Phytochemical composition in *R. fruticosus* varies depending on growing conditions and environmental factors [23,28]. Different *Rubus* species exhibit varying profiles of organic acids, with *R. fruticosus* typically exhibiting high malic acid content [45,46]. Other organic acids contributing to the functional properties of blackberries include citric, fumaric, and oxalic acids [47]. Moreover, blackberries are rich in essential minerals including potassium, calcium, magnesium, and iron—as well as vitamins C and K, which contribute to a nutritionally balanced diet [48–50].

Despite increasing global research on blackberries, comprehensive studies integrating morphological, biochemical, and phytochemical diversity of *R. fruticosus* populations remain limited, particularly in South Asia. In Pakistan, *R. fruticosus* has traditionally served important ecological and economic functions, particularly as protective hedges for high-value crops such as apricots and apples in Khyber Pakhtunkhwa. However, habitat degradation, replacement of natural hedges with barbed wire fencing, and the introduction of hybrid varieties for aesthetic purposes have collectively endangered local landraces [51,52]. No integrated study combining morphological characterization, SDS-PAGE-based seed protein profiling, and HPLC-UV phytochemical analysis currently exists for *R. fruticosus* landraces from Khyber Pakhtunkhwa. Therefore, the present study was designed to assess the morpho-biochemical diversity and quantify major bioactive compounds including phenolic acids, anthocyanins, and vitaminsin native *R. fruticosus* landraces. We hypothesize that significant morpho-biochemical and phytochemical variation exists among local landraces, reflecting underlying genetic diversity and environmental adaptation. This study aims to provide an integrated evaluation of their nutritional and therapeutic potential and identifying superior genotypes for future breeding programs and applications in the food and pharmaceutical industries. To the best of our knowledge, this represents the first comprehensive study simultaneously integrating morphological characterization, SDS-PAGE-based seed protein profiling, and HPLC-UV phytochemical analysis for *R. fruticosus* landraces from the Malakand Division, Khyber Pakhtunkhwa, thereby addressing a critical gap in South Asian germplasm research and providing baseline data essential for informed conservation planning, pre-breeding, and molecular studies.

## Materials and methods

### Ethical approval and field permits

The current study was conducted in compliance with applicable institutional, national, and international guidelines for field collection of plant material. Ethical approval was granted by the Institutional Ethics Committee of the University of Malakand. Collection of plant material from the study sites did not require special government permits, as the sampled areas constitute non-protected forest zones in the Malakand Division; however, all collections adhered to local land-use regulations. Voucher specimens have been deposited at the Herbarium of the Department of Botany, University of Malakand (Voucher Nos. UOM-BOT-2025-RF-01 to RF-15). All landraces were represented by five individual plants (n = 5 per site) to ensure biological replication across all analyses.

### Study area and plant collection

**Fig 1:**
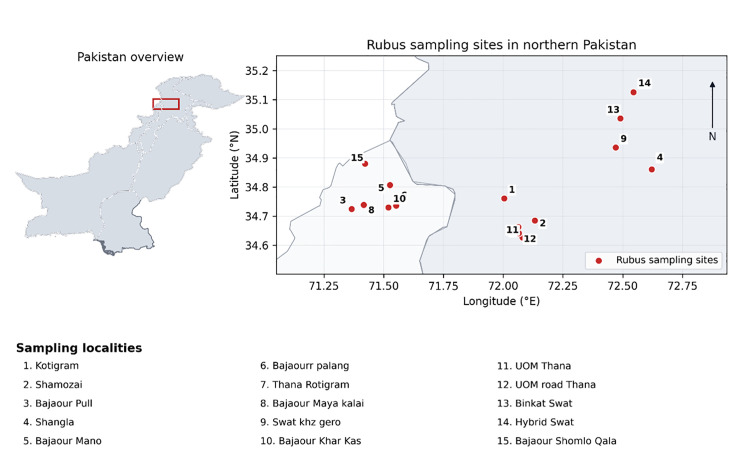
Map of sampling sites.

The present study was conducted at the Department of Botany, University of Malakand, Chakdara, Dir (Lower), Khyber Pakhtunkhwa, Pakistan, from July 2024 to October 2025. The Malakand Division in northern Khyber Pakhtunkhwa has a warm temperate climate influenced by the southwest monsoon and winter western disturbances [53]. The mean annual temperature ranges between 19.9 and 22 °C, with summer temperatures reaching up to 31 °C [53,54]. Annual precipitation ranges from 1000 to 1500 mm [55]. The soils are predominantly sandy loam with silty loam and loamy sand textures and are slightly alkaline to neutral (pH 6.7–8.2) [56]. A total of fifteen indigenous landraces of *Rubus fruticosus* L. were collected from fifteen sites (S1 Table and Fig 1) during exploratory field surveys carried out across ecologically distinct zones of the Malakand Division, including Dir (Lower), Dir (Upper), Swat, Buner, and Malakand Agency, encompassing sites at varying altitudes (800–2,400 m above sea level (Fig 1). The fifteen landraces and their collection localities are listed in S1 Table. Collection was performed during the fruiting season (July–September 2024). For each landrace, plant material including fruits, leaves, stems, and seeds was systematically harvested from a minimum of five representative individuals per site. Geographic coordinates (GPS), altitude, and basic habitat descriptors (vegetation type, slope aspect, land use) were recorded at each collection site using a Garmin eTrex handheld GPS device. All GPS coordinates, altitude data, and habitat descriptors for each site are provided in S1 Table. All plant specimens were identified to species level by Dr. Mohammad Nisar, a taxonomic botanist at the Department of Botany, University of Malakand, using established regional flora keys. The identity of each accession as *Rubus fruticosus* L. was confirmed by comparison with authenticated herbarium specimens deposited at the University of Malakand Herbarium (voucher accession numbers UOM-BOT-2024-RF-01 to RF-07). The study area map (Fig 1) was generated using R statistical software with the ggplot2, ggspatia and sf packages.

### Morphological characterization

Morphological data were recorded according to the descriptors established by the International Board for Plant Genetic Resources [57] and subsequent compilations [58]. A total of seventeen traits were assessed, comprising eight quantitative traits—plant height, number of branches per plant, leaflet number, leaf width, leaf length, internode length, single fruit weight, and five-fruit weight—and nine qualitative traits—stem type, stem color, leaf upper surface color, leaf lower surface color, leaf margin, leaf arrangement, leaf venation, fruit color, and flower color. All measurements were performed at the appropriate phenological stage. Plant height was recorded in centimeters (cm) using a measuring tape, while all leaf, internode, and fruit measurements were conducted with digital Vernier calipers. Qualitative traits were scored following standardized descriptor scales. Observations were conducted on at least five plants per landrace. The study was laid out in a randomized complete block design (RCBD) with three replications.

### Seed protein profiling by SDS-PAGE

Total seed protein profiles of the fifteen *Rubus* landraces were assessed by sodium dodecyl sulfate–polyacrylamide gel electrophoresis (SDS-PAGE) following the protocol described by Nisar *et al*., [59]. Mature, healthy seeds were ground to a fine powder using a mortar and pestle, and 0.02 g of powdered material was transferred to 1.5 mL microcentrifuge tubes. Each sample was homogenized with 400 µL of protein extraction buffer (0.05 M Tris-HCl, pH 8.0; 0.2% SDS; 5 M urea; 1% 2-mercaptoethanol) by vortexing for 1 min and subsequently centrifuged at 12,000 rpm for 10 min at room temperature. The supernatant was collected and used for electrophoresis. Proteins were resolved on 15% polyacrylamide gels comprising a separating gel (20% acrylamide, 0.135% N, N-methylene-bisacrylamide, 0.15 M Tris-HCl pH 8.8, 0.27% SDS) and a stacking gel. Tetramethylethylenediamine (TEMED) was added last to initiate polymerization. A standard molecular weight marker (14.4–97.4 kDa range) was included in each gel run for band size estimation. Following electrophoresis, gels were stained with Coomassie Brilliant Blue R-250, and band patterns were recorded using a gel documentation system. Each experiment was independently replicated three times to ensure reproducibility. Band presence was scored as 1 and absence as 0 to construct a binary data matrix [44].

### Extract preparation and HPLC-UV phytochemical profiling

The seven representative landraces exhibiting the greatest morphological divergence based on preliminary morphological analysis and were processed for phytochemical profiling in order to represent the biochemical variability within the sampled populations. Methanolic extracts of *R. fruticosus* fruits were prepared following the method described by Zeb [60]. Approximately 1.0 g of freeze-dried, powdered fruit sample was combined with 20 mL of a methanol–water mixture (1:1, v/v) in a conical flask. The mixture was heated in a thermostatic water bath at 70 °C for 60 min, then centrifuged at 4,000 rpm for 10 min. A 2 mL aliquot of the resultant supernatant was passed through Whatman No. 1 filter paper and transferred into an HPLC vial.

HPLC analysis was performed using an Agilent 1260 Infinity system equipped with an Agilent Zorbax Eclipse XDB-C18 column (150 mm × 4.6 mm, 5 µm particle size). Gradient elution was carried out using two mobile phases: Solvent A (methanol: acetic acid: deionized water, 100:20:180 v/v) and Solvent B (methanol: acetic acid: deionized water, 900:20:80 v/v). The gradient program was as follows: 0–5 min, 5% B; 5–20 min, 5–50% B; 20–30 min, 50–80% B; 30–35 min, 80% B; 35–40 min, 80–5% B, followed by 5 min re-equilibration at initial conditions. The flow rate was set at 1.0 mL/min, the injection volume was 10 µL, and the column temperature was maintained at 30 °C. Detection was performed using a diode-array detector (DAD) set at 320 nm, targeting phenolic compounds. Phenolic compounds were identified by comparison of retention times and UV spectral profiles with certified external standards. Quantification was performed using the external standard method according to the formula: C_x_ = (A_x_/ A_s_) × C_s_, where C_x_ is the sample concentration (µg/mL), A_x_ is the sample peak area, A_s_ is the standard peak area, and C_s_ is the standard concentration (0.09 µg/mL). All extractions and chromatographic analyses were performed in triplicate to ensure reproducibility.

### Statistical analysis

Quantitative and qualitative morphological data from the seven landraces were analyzed using multiple statistical approaches. Frequency distributions for qualitative traits were computed in Microsoft Excel 2016. Quantitative data were subjected to descriptive statistics including range, mean, maximum, minimum, variance, standard deviation, standard error, and coefficient of variation (CV%)using IBM SPSS Statistics version 22.0. Pearson correlation coefficients among quantitative traits were computed using the same software. All tests were evaluated at a significance level of p < 0.05. Data were checked for normality prior to analysis using the Shapiro–Wilk test. Prior to PCA, all quantitative variables were standardized using z-score normalization to ensure equal weighting of variables with different measurement scales. PCA and hierarchical cluster analysis (HCA) were performed using Ward’s method with Euclidean distance in PC-ORD version 6.0. For SDS-PAGE analysis, band presence/absence data were encoded in a binary matrix, and two-way cluster dendrograms were constructed in PC-ORD version 6.0. Shannon’s diversity index (H′) was calculated for each SDS-PAGE locus using standard formula.

## Results

The seven land races of *R. fruticosus* collected from fifteen ecologically distinct zones of the Malakand Division were systematically evaluated for morphological, biochemical, and phytochemical traits. Morphological characterization of seven *R. fruiticosus* landraces revealed substantial phenotypic variation across qualitative and quantitative traits. S1 Table lists the seven landraces evaluated in this study, together with their collection localities, altitudinal ranges, and geographic coordinates (Fig 1). Both qualitative and quantitative morphological traits exhibited considerable inter-landrace variability, as detailed below.

### Qualitative traits

With respect to qualitative traits (Table 1), stem type was variable, with 60% of genotypes displaying a prostrate growth habit and 40% an erect form. Stem color also differed markedly: 73% of genotypes exhibited green stems, while 27% were reddish. Leaf surface traits showed notable diversity; the upper surface was dark green in 60% and green in 40% of genotypes, while the lower surface was green in 53% and light green in 47%. Leaf margin varied across serrate (46%), toothed (27%), and saw-toothed (27%) morphotypes. Leaf arrangement was alternate in 53% and opposite in 47% of genotypes. Leaf venation was equally distributed among reticulate, net-veined 28%, and netted types (43%). All genotypes produced black fruits at maturity. Flower color showed variation, with 57% of genotypes producing pink flowers and 43% white flowers.

**Table 1:** Distribution of qualitative morphological traits among the studied *Rubus fruticosus* genotypes.

Trait Category	Trait	Phenotypic Class	Number of Genotypes (n = 7)	Frequency (%)
Stem	Stem type	Prostrate	4	57.1
		Erect	3	42.9
	Stem color	Green	5	71.4
		Reddish	2	28.6
Leaf	Leaf surface (upper)	Dark green	4	57.1
		Green	3	42.9
	Leaf surface (lower)	Green	4	57.1
		Light green	3	42.9
	Leaf margin	Serrate	3	42.9
		Toothed	2	28.6
		Saw-toothed	2	28.6
	Leaf arrangement	Alternate	4	57.1
		Opposite	3	42.9
	Leaf venation	Reticulate	2	28.6
		Net-veined	2	28.6
		Netted	3	42.9
Fruit	Fruit color	Black	7	100
		Pink	4	57.1
		White	3	42.9

### Quantitative traits

Significant variation was observed among quantitative morphological traits (Table 2). Plant height ranged from 2.58 to 8.00 ft, with a mean of 3.94 ft (CV = 31.31%). The number of branches per plant varied from 4.00 to 12.90, with a mean of 7.43 (CV = 33.33%). Leaflet number ranged from 3.20 to 5.00, averaging 4.04 (CV = 12.44%). Leaf width ranged from 1.84 to 5.70 cm (mean = 3.61 cm; CV = 27.85%), while leaf length ranged from 3.32 to 7.40 cm (mean = 5.34 cm; CV = 21.17%). Internode length varied from 3.34 to 8.84 cm (mean = 5.00 cm; CV = 30.11%). Single fruit weight ranged from 0.27 to 2.10 g (mean = 0.59 g; CV = 73.16%), and five-fruit weight varied from 1.30 to 8.53 g (mean = 2.64 g; CV = 64.38%). The highest morphological variability was thus recorded for fruit-related traits, followed by vegetative growth traits, while leaflet number was the most stable character (CV = 12.44%).

**Table 2:** Descriptive statistics of different quantitative traits of *Rubus Fruiticosus* genotypes.

S/No	Traits	Mean	Standard Error	Standard Deviation	Sample Variance	Range	Minimum	Maximum	CV% (AVA: 36.72
1	Plant Height (ft)	3.94	0.32	1.23	1.52	5.42	2.58	8.00	31.31
2	Branches	7.43	0.64	2.48	6.14	8.90	4.00	12.90	33.33
3	Leaflet Number	4.04	0.13	0.50	0.25	1.80	3.20	5.00	12.44
4	Leaf width (cm)	3.61	0.26	1.01	1.01	3.86	1.84	5.70	27.85
5	Leaf length (cm)	5.34	0.29	1.13	1.28	4.08	3.32	7.40	21.17
6	Internode length (cm)	5.00	0.39	1.51	2.27	5.50	3.34	8.84	30.11
7	Single Fruit weight (g)	0.59	0.11	0.43	0.19	1.83	0.27	2.10	73.16
8	5 fruit weight (g)	2.64	0.44	1.70	2.90	7.23	1.30	8.53	64.38

### Comparative analysis of quantitative traits across genotypes

**Fig 2:**
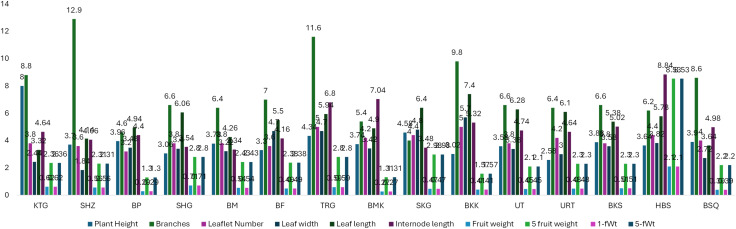
Comparative chromatogram of different quantitative traits of *Robus* genotypes.

Fig 2 illustrates variation in quantitative traits across the fifteen *R. fruticosus* genotypes. Among all genotypes, Hybrid Swat (HBS) recorded the tallest plant height (8.00 ft) and the highest single fruit weight (2.10 g) and five-fruit weight (8.53 g), while Uom road Thana (URT) exhibited the shortest stature (2.58 ft). Branch number was highest in Shamozai (SHZ) (12.90) and lowest in Swat Khz gero (SKG) (4.00). Bajaour Khar Kas (BKK) recorded the greatest leaf width (5.70 cm) and leaf length (7.40 cm), whereas Shamozai (SHZ) showed the narrowest leaves (1.84 cm) and Kotigram (KT) the shortest leaves (3.32 cm). Internode length was greatest in Hybrid Swat (HBS) (8.84 cm) and shortest in Bajaour Mano (BM) (3.34 cm). Collectively, genotypes Hybrid Swat (HBS) and Bajaour Khar Kas (BKK) exhibited superior agronomic performance, particularly with respect to fruit yield and vegetative development, whereas Uom road Thana (URT), Swat Khz gero (SKG), and Bajaour Pull (BP) showed comparatively lower performance Variation across genotypes likely reflects environmental heterogeneity across collection sites, including altitude and habitat differences.

### Pearson correlation analysis of quantitative traits

**Fig 3:**
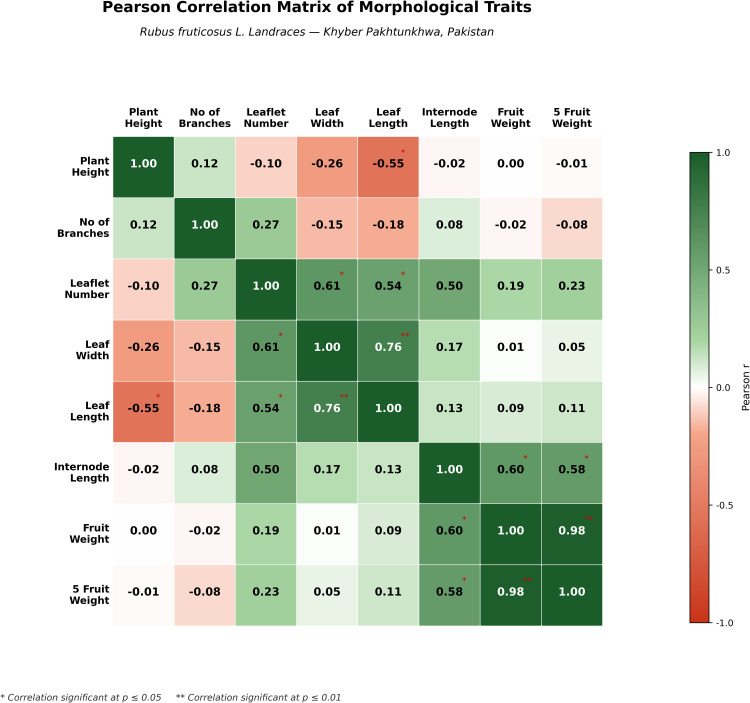
Pearson correlation analysis of morphological parameters of *Rubus fruticosus.*

Pearson correlation analysis among quantitative morphological traits revealed several statistically significant associations (Fig 3; p < 0.05). Leaf width was positively correlated with leaflet number (*r* = 0.61*). Leaf length exhibited a significant negative correlation with plant height (r = −0.55*), but positive correlations with leaflet number (*r* = 0.54*) and leaf width (*r* = 0.76**). Single fruit weight showed a significant positive correlation with internode length (r = 0.60*), and five-fruit weight was positively correlated with both internode length (*r* = 0.58*) and single fruit weight (*r* = 0.98**). These correlation patterns indicate interdependence among morphological traits, particularly between vegetative and reproductive yield parameters, and informative for indirect selection strategies in breeding programs.

### Principal component analysis

**Fig 4:**
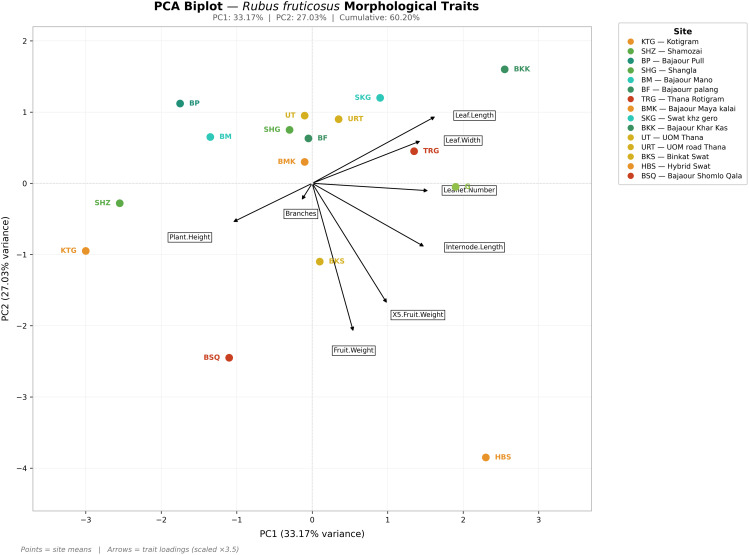
PCA biplots of morphological parameters of *Rubus fruticosus.*

PCA of the eight quantitative morphological traits explained a cumulative variance of 60.20% across two principal components (Fig 4). PC1 accounted for 33.17% of the total variance and was predominantly characterized by positive loadings for Leaf length, leaf width, and leaflet number. PC2 explained 27.03% of the variance and was influenced negatively by plant height with PC1. The PCA biplot (Fig 4) revealed distinct separation based on morphological trait associations. Leaf Length and Leaf Width vectors projected toward the upper-right quadrant, with BKK and SKG align closely with these traits, indicating superior leaf morphology. Internode Length and X5 Fruit Weight vectors projected toward the lower-right, with HBS positioned in this region, reflecting distinct fruit and stem morphological characteristics. Fruit Weight projected toward the lower-left, where BSQ and BKS clustered, indicating enhanced fruit weight traits. Plant Height projected toward the far left, with KT and SHZ reflecting divergent vegetative stature. The Branches vector associated with BP and BM in the upper-left quadrant denotes distinct branching characteristics. The remaining collection sites, including UT, URT, SHG, BF, BMK, and TRG, clustered near the biplot origin, reflect moderate and overlapping morphological variation among *R. fruticosus* landraces in the study region.PCA revealed that fruit yield-related traits and vegetative growth parameters were the major contributors to phenotypic variation among *R. fruticosus* genotypes, indicate their importance in selection and breeding programs**.**

### Hierarchical cluster analysis

**Fig 5:**
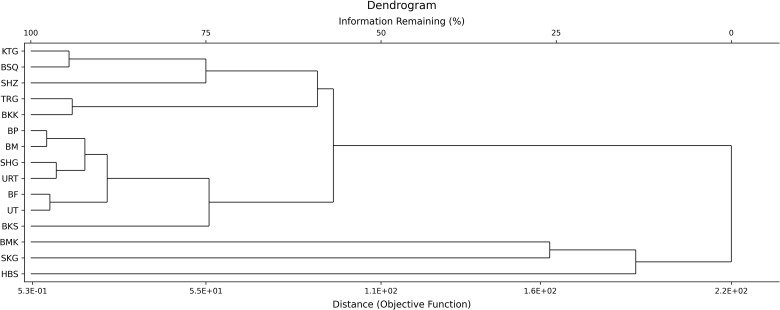
Cluster analysis of morphological traits of Robus genotypes collected from different ecological zones.

Hierarchical cluster analysis was performed in PC-ORD version 6.0 using Ward’s minimum variance method with Euclidean distance as the dissimilarity measure (Fig 5). At a phenotypic distance of 25%, all Fifteen genotypes were divided into two major lineages, which were further subdivided into five clusters at a dissimilarity threshold of 75% (Fig 5). Genotypes (KT), and (HBS), were the most divergent, each occupying distinct positions within the dendrogram. The first subgroup comprised two genotypes (KT) and (BSQ); the second subgroup consisted of one genotype (SHZ); the third subgroup contained two genotypes (TRG) and (BKK); the fourth subgroup—the largest—comprised six genotypes (BP), (SHG), (URT), (BF), (UT), and (BKS); and the fifth subgroup consisted of a single genotype (HBS). Within each cluster, genotypes exhibited relatively low inter-individual variation compared with the divergence observed between clusters, reflecting the influence of ecological zone and geographic collection site on morphological differentiation.

### Seed protein diversity by SDS-PAGE

**Fig 6:**
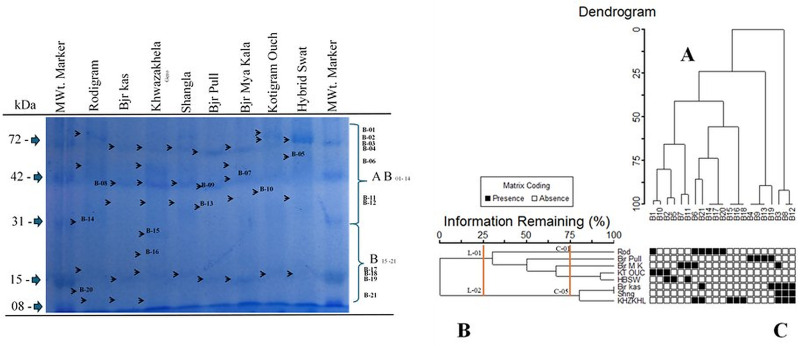
Two-way cluster analysis of total seed storage proteins profile of 08 genotypes of *Rubus fruiticosus.* A: Cluster pattern of 21 polypeptide bands distributed in collected genotypes. B: cluster analysis of 08 genotypes based on genetic distance across the polypeptide bands. C: Zymogram of 21 bands distributed in 08 genotypes. At 25% and 75% genetic similarity the cluster plot was divided into 2 lineages and 05 clusters respectively.

SDS-PAGE analysis of seed storage proteins from the Eight *R. fruticosus* landraces resolved a total of 21 scorable bands within the molecular weight range of 14.4–97.4 kDa, as determined using a standard protein marker. All 21 bands were polymorphic across the genotype panel, demonstrating a high degree of biochemical diversity. The electrophoretic profiles exhibited clear, well-resolved, and reproducible banding patterns across all genotypes (6C) (zymogram), ensuring reliable band scoring and accurate assessment of protein polymorphism**.** Shannon’s diversity index (H′) and the coefficient of variation (CV%) were calculated for each locus to quantify inter-genotype variation (Table 3). Considerable variability was observed across loci: the highest CV values were recorded for markers B-03 (93.54%), B-06, B-08, B-12, and B-21 (each approximately 72.72%), indicating high dispersion and substantial discriminatory utility. In contrast, several markers exhibited relatively lower CV values (~35.36%), reflecting moderate cross-genotype consistency. Shannon’s H′ values ranged from 0.00 to 1.39, with B-03 recording the highest diversity (H′ = 1.39), whereas markers B-04, B-09, and B-13 to B-16 exhibited uniform banding patterns across all genotypes (H′ = 0.00). Two-way hierarchical cluster analysis of the binary protein data segregated the genotypes into two major lineages and five sub-clusters (Fig 6), with genotypes “(TRG)” and “(SKG)” appearing at the dendrogram periphery, reflecting the greatest divergence in seed protein composition.

**Table 3:** Percent coefficient of variation and Shannon diversity index in *Rubus fruiticosus* seed protein profile using SDS-PAGE.

	N	Min	Max	Mean	Std. error	Stand. dev	CV %age	Shanno-H Index
B-01	8	0	1	0.25	0.16	0.46	54.01	0.69
B-02	8	0	1	0.25	0.16	0.46	54.01	0.69
B-03	8	0	1	0.50	0.19	0.53	93.54	1.39
B-04	8	0	1	0.13	0.13	0.35	35.36	0.00
B-05	8	0	1	0.13	0.13	0.35	35.36	0.00
B-06	8	0	1	0.38	0.18	0.52	72.46	1.10
B-07	8	0	1	0.13	0.13	0.35	35.36	0.00
B-08	8	0	1	0.38	0.18	0.52	72.46	1.10
B-09	8	0	1	0.13	0.13	0.35	35.36	0.00
B-10	8	0	1	0.13	0.13	0.35	35.36	0.00
B-11	8	0	1	0.25	0.16	0.46	54.01	0.69
B-12	8	0	1	0.38	0.18	0.52	72.46	1.10
B-13	8	0	1	0.13	0.13	0.35	35.36	0.00
B-14	8	0	1	0.13	0.13	0.35	35.36	0.00
B-15	8	0	1	0.13	0.13	0.35	35.36	0.00
B-16	8	0	1	0.13	0.13	0.35	35.36	0.00
B-17	8	0	1	0.13	0.13	0.35	35.36	0.00
B-18	8	0	1	0.13	0.13	0.35	35.36	0.00
B-19	8	0	1	0.25	0.16	0.46	54.01	0.69
B-20	8	0	1	0.13	0.13	0.35	35.36	0.00
B-21	8	0	1	0.38	0.18	0.52	72.46	1.10

### Phytochemical profiling by HPLC-UV

Owing to instrument availability and resource constraints, HPLC-UV phytochemical profiling was conducted on seven representative genotypes purposively selected to encompass the full spectrum of morphological and biochemical diversity identified in preceding analyses—specifically, genotypes spanning both ends of the PCA ordination and the two major SDS-PAGE clusters (Thana Rotigram(TRG), (Bajaour Khar Kas(BKK), (Swat Khz gero(SKG), Bajaour Pull(BP), (Hybrid Swat(HBS), Binkat Swat(BKS), and Shangla(SHG). Methanolic extracts of these seven *R. fruticosus* genotypes were subjected to HPLC-UV analysis for phenolic compound identification and quantification (Tables 4 and 5). A total of twelve phenolic compounds were identified across the analyzed genotypes based on comparison with certified external standards. Notable intergenotype variability was observed in both the presence and quantitative levels of individual compounds. Morin concentrations were highest in genotype (Thana Rotigram(TRG) (3,945.18 µg/mL peak area equivalent; 13.85%), while quercetin was most abundant in (Swat Khz gero(SKG) (8,993.06 µg/mL equivalent; 16.35%). Certain compounds—including ellagic acid, catechin hydrate and caffeic acid—were entirely absent in specific genotypes, further substantiating genetic variation in secondary metabolite biosynthesis.

**Table 4:** Identification of phenolics compounds using HPLC analysis.

Genotypes	RT	Compound	Formula	Mol Weight (g/mol)	Peak area	Area %
Kotidigram (KT)	2.374	Malic acid	C4H6O5	134.087	81.99	0.28
	4.325	Gallic acid	C7H6O5	170.12	25.49	0.08
	5.054	Vitamin C	C6H8O6	176.124	320.59	1.12
	6.134	Chlorogenic acid	C16H18O9	354.31	216.35	0.75
	9.247	Epigalactocatechin gallate	C22H18O11	458.372	2902.88	10.19
	10.810	Quercetin	C15H10O7	302.236	94.80	0.33
	12.297	Morin	C15H10O7	302.235	3945.18	13.85
	16.100	Ellagic acid	C14H6O8	302.197	1042.77	3.66
	20.826	Catechin hydrate	C15H14O6	290.26	375.51	1.31
	29.515	Caffeic acid	C9H8O4	180.16	33.40	0.11
	35.208	Phloroglucinol	C6H6O3	126.11	88.52	0.31
	37.462	Hydroxyl benzoic acid	C7H6O3	138.12	242.47	0.85
Bjr Kas	2.604	Malic acid	C4H6O5	134.087	664.48	1.15
	4.061	Gallic acid	C7H6O5	170.12	28.37	0.04
	5.070	Vitamin C	C6H8O6	176.124	2101.52	3.64
	6.288	Chlorogenic acid	C16H18O9	354.31	563.91	0.97
	9.239	Epigalactocatechin gallate	C22H18O11	458.372	4790.63	8.31
	10.820	Quercetin	C15H10O7	302.236	2813.55	4.88
	12.008	Morin	C15H10O7	302.235	43.51	0.07
	16.528	Ellagic acid	C14H6O8	302.197	47.75	0.08
	20.872	Catechin hydrate	C15H14O6	290.26	3081.43	5.34
	30.680	Caffeic acid	C9H8O4	180.16	128.13	0.22
	35.649	Phloroglucinol	C6H6O3	126.11	696.44	1.20
	37.547	Hydroxyl benzoic acid	C7H6O3	138.12	229.46	0.39
Khwaza khela Gero	2.618	Malic acid	C4H6O5	134.0874	485.19	0.88
	4.571	Gallic acid	C7H6O5	170.12	711.36	1.29
	5.085	Vitamin C	C6H8O6	176.124	1861.04	3.38
	6.290	Chlorogenic acid	C16H18O9	354.31	771.61	1.40
	9.273	Epigalactocatechin gallate	C22H18O11	458.372	5432.00	9.87
	10.009	Quercetin	C15H10O7	302.236	8993.06	16.35
	12.056	Morin	C15H10O7	302.2357	119.06	0.21
	16.228	Ellagic acid	C14H6O8	302.197	112.23	0.20
	20.944	Catechin hydrate	C15H14O6	290.26	2670.20	4.85
	30.714	Caffeic acid	C9H8O4	180.16	185.24	0.33
	35.683	Phloroglucinol	C6H6O3	126.11	807.61	1.46
	37.599	Hydroxyl benzoic acid	C7H6O3	138.12	224.84	0.40
Bjr Khar pull	2.327	Malic acid	C4H6O5	134.08	43.51	0.31
	4.774	Gallic acid	C7H6O5	170.12	33.20	0.24
	5.064	Vitamin C	C6H8O6	176.12	48.50	0.35
	6.143	Chlorogenic acid	C16H18O9	354.31	371.84	2.71
	9.126	Epigalactocatechin gallate	C22H18O11	458.37	51.20	0.37
	10.779	Quercetin	C15H10O7	302.23	349.77	2.55
	12.273	Morin	C15H10O7	302.23	1863.60	13.60
	16.001	Ellagic acid	C14H6O8	302.19	33.82	0.24
	30.657	Caffeic acid	C9H8O4	180.16	177.07	1.29
	35.237	Phloroglucinol	C6H6O3	126.11	87.99	0.64
	36.260	Hydroxyl benzoic acid	C7H6O3	138.12	308.28	2.24
Hybrid swat (HBS)	2.604	Malic acid	C4H6O5	134.08	664.48	1.15
	4.061	Gallic acid	C7H6O5	170.12	28.37	0.04
	5.070	Vitamin C	C6H8O6	176.12	2101.52	3.64
	6.288	Chlorogenic acid	C16H18O9	354.31	563.91	0.97
	9.239	Epigalactocatechin gallate	C22H18O11	458.37	4790.63	8.31
	10.820	Quercetin	C15H10O7	302.23	2813.55	4.88
	12.008	Morin	C15H10O7	302.23	43.51	0.07
	16.528	Ellagic acid	C14H6O8	302.19	47.75	0.08
	20.872	Catechin hydrate	C15H14O6	290.26	3081.43	5.34
	30.680	Caffeic acid	C9H8O4	180.16	128.13	0.22
	35.249	Phloroglucinol	C6H6O3	126.11	69.15	0.12
	36.281	Hydroxyl benzoic acid	C7H6O3	138.12	313.69	0.54
Binkat Swat (BKS)	2.611	Malic acid	C4H6O5	134.08	366.65	2.49
	4.785	Gallic acid	C7H6O5	170.12	50.74	0.34
	5.112	Vitamin C	C6H8O6	176.12	179.14	1.21
	6.167	Chlorogenic acid	C16H18O9	354.31	102.56	0.69
	8.059	Epigalactocatechin gallate	C22H18O11	458.37	68.69	0.46
	9.258	Quercetin	C15H10O7	302.23	418.44	2.84
	12.445	Morin	C15H10O7	302.23	1079.72	7.33
	20.887	Catechin hydrate	C15H14O6	290.26	120.19	0.81
	30.653	Caffeic acid	C9H8O4	180.16	132.31	0.89
	35.192	Phloroglucinol	C6H6O3	126.11	90.72	0.61
	36.207	Hydroxyl benzoic acid	C7H6O3	138.12	310.72	2.11
Shangla (SHG)	2.604	Malic acid	C4H6O5	134.08	664.48	1.15
	4.061	Gallic acid	C7H6O5	170.12	28.37	0.04
	5.070	Vitamin C	C6H8O6	176.12	2101.52	3.64
	6.288	Chlorogenic acid	C16H18O9	354.31	563.91	0.97
	8.077	Epigalactocatechin gallate	C22H18O11	458.37	824.30	1.43
	9.239	Quercetin	C15H10O7	302.23	4790.63	8.31
	12.008	Morin	C15H10O7	302.23	43.51	0.07
	16.528	Ellagic acid	C14H6O8	302.19	47.75	0.08
	20.872	Catechin hydrate	C15H14O6	290.26	3081.43	5.34
	35.249	Phloroglucinol	C6H6O3	126.11	69.15	0.12
	37.547	Hydroxyl benzoic acid	C7H6O3	138.12	229.46	0.39

**Table 5:** Comparative distribution of phenolic compounds in 07 landraces of *Rubus fruiticosus* collected from Malakand division using HPLC-UV analysis.

S/No	R. Fru-01 Binkat Swat	R. Fru-02 Rodigrame	R. Fru-05 Bajaour Pull	R. Fru-12 Bajaour Khar Kas	R. Fru-12a Hybrid Swat	R. Fru-14 Swat Khza Gero	R. Fru-12 b Shangla
1	Malic acid	Malic acid	Malic acid	Malic acid	Malic acid	Malic acid	Malic acid
2	Gallic acid	Gallic acid	Gallic acid	Gallic acid	Gallic acid	Gallic acids	Gallic acids
3	Vitamin C	Vitamin C	Vitamin C	Vitamin C	Vitamin C	Vitamin C	Vitamin C
4	Chlorogenic acid	Chlorogenic acid	Chlorogenic acid	Chlorogenic acid	Chlorogenic acid	Chlorogenic acid	Chlorogenic acid
5	Epigalactocatechin gallate	Epigalactocatechin gallate	Epigalactocatechin gallate	Epigalactocatechin gallate	Epigalactocatechin gallate	Epigalactocatechin gallate	Epigalactocatechin gallate
6	Quercetin	Quercetin	Quercetin	Quercetin	Quercetin	Quercetin	Quercetin
7	Morin	Morin	Morin	Morin	Morin	Morin	Morin
8	**Not detected**	Ellagic acid	Ellagic acid	Ellagic acid	Ellagic acid	Ellagic acid	Ellagic acid
9	Catechin hydrate	Catechin hydrate	**Not detected**	Catechin hydrate	Catechin hydrate	Catechin hydrate	Catechin hydrate
10	Caffeic acid	Caffeic acid	Caffeic acid	Caffeic acid	Caffeic acid	Caffeic acid	**Not detected**
11	Phloroglucinol	Phloroglucinol	Phloroglucinol	Phloroglucinol	Phloroglucinol	Phloroglucinol	Phloroglucinol
12	Hydroxyl benzoic acid	Hydroxyl benzoic acid	Hydroxyl benzoic acid	Hydroxyl benzoic acid	Hydroxyl benzoic acid	Hydroxyl benzoic acid	Hydroxyl benzoic acid

### Functional relevance of identified phenolic compounds

The twelve phenolic compounds identified in this study (Table 6) are well-established bioactive constituents with documented antioxidant, anti-inflammatory, antimicrobial, and chemopreventive properties. Quercetin is a flavonoid with broad therapeutic relevance, including anti-inflammatory and antioxidant activities [61]. Epigallocatechin gallate is associated with metabolic syndrome prevention and neuroprotective effects [62]. Morin exhibits potent radical scavenging activity attributable to its catechol-type structure [63]. Ellagic acid possesses antiproliferative and antioxidant properties relevant to nutraceutical applications [24]. Caffeic acid, chlorogenic acid, gallic acid, catechin hydrate, and phloroglucinol are additional phenolics with well-characterized biological functions [64–68]. The identification of benzoic acid derivatives is also noteworthy, given their roles in antimicrobial activity [69]. The collective presence of these compounds in native *R. fruticosus* landraces underscores their substantial nutraceutical and pharmaceutical value.

**Table 6:** The possible function of 12 phenolic compounds detected in 07 landraces of *Rubus fruiticosus* collected from the Malakand division using HPLC-UV analysis.

S/No	Phenolic Compounds	Possible Function	Reference
1	Malic acid	food and beverage industry, low transition temperature mixtures,	Kövilein et al., 2020
2	Gallic acid	Antioxidant capacity, anticancer, anti-HIV, antiulcerogenic, anti-inflammatory	Fernandes & Salgado, 2016
3	Vitamin C	Skeletal muscle, lipid peroxidation byproducts, muscle injury	Evans, 2000
4	Chlorogenic acid	coffee and tea, antioxidant agents	Naveed et al., 2018
5	Epigalactocatechin gallate	cardiovascular events, insulin resistance, high blood pressure, and dyslipidemia.	Legeay et al., 2015
6	Quercetin	Biosynthesis and regulation, signal transduction, plant stress tolerance.	Singh et al., 2021
7	Morin	Inhibit lipid peroxidation, deactivate free radical generating enzymes,	Mendoza-Wilson et al., 2011
8	Ellagic acid	beneficial health effects against oxidation-linked chronic diseases such as cancer and cardiovascular diseases, effects of oxidative stress by directly acting as an antioxidant	Vattem & Shetty, 2005
9	Catechin hydrate	antioxidative and anti-inflammatory properties, neuroprotective and anti-cancer effects.	Kaur et al., 2017
10	Caffeic acid	Play role in defense mechanism of plants against predators, and also have inhibitory effect on the growth of insects, fungi and bacteria	Espíndola et al., 2019
11	Phloroglucinol	low toxic smooth muscle relaxants, possess broad spectrum antiviral, antibacterial, antifungal, antihelminthic and phytotoxic properties.	Yang & Cao, 2012
12	Hydroxyl benzoic acid	Acting as a recalcitrant organic pollutant in wastewater, Contributing to toxicity and persistence in industrial effluents.	Wu et al., 2017

## Discussion

The present study provides a comprehensive assessment of morphological, biochemical, and phytochemical diversity among fifteen *R. fruticosus* landraces collected from distinct ecological zones of the Malakand Division, Khyber Pakhtunkhwa. The integration of morphological descriptors, SDS-PAGE-based seed protein profiling, and HPLC-UV phytochemical analysis yielded robust insights into the genetic and functional variability of this understudied germplasm.

The landraces exhibited marked diversity in the qualitative morphological traits (Table 1). stem growth habit, which were either prostrate or erect, reflect the influence of genetic and environmental factors [70]. The color of the stems and leaves is likely associated with the levels of flavonoids and anthocyanins [71]. There was variability in leaf characteristics such as margin, arrangement, and venation due to their taxonomic significance [70]. There was genetic variation in reproductive traits, as flower color was varied between pink and white [72], However, fruit color was uniformly black [71]. These qualitative traits, combined with the quantitative ones further highlight the overall diversity of the germplasm.

The extensive quantitative morphological variability documented in this study is consistent with the recognized genetic plasticity of *Rubus* species across diverse environmental gradients. Significant inter-genotype differences were observed for plant height, branch number, leaf dimensions, internode length, and fruit-related traits. Genotypes (Hybrid Swat (HBS) and Bajaour Khar Kas (BKK) exhibited superior agronomic performance with respect to fruit yield and vegetative growth, while (UOM road Thana (URT), Swat khz gero (SKG), and (Bajaour Pull (BP) showed consistently lower values across most measured characters (Table 2). This pattern of diversity may reflect both intrinsic genetic variation and phenotypic plasticity in response to site-specific environmental factors, including altitude, soil type, moisture availability, and temperature—all of which are known to influence *Rubus* phenotype [23,28]. The findings are in agreement with previous reports of substantial phenotypic diversity in *Rubus* species [26,41,73]and corroborate the utility of morphological traits as primary descriptors for germplasm evaluation. (Fig 2), which is the graphical representation of the quantitative traits, substantiates the variability recorded in (Table 2) by depicting the differences between genotypes. Additionally, the hierarchical cluster analysis (Fig 5) using the same quantitative traits classified the genotypes into separate clusters, which indicated the similarity and difference in their morphology. The finding of extremely divergent genotypes like KT and HBS emphasizes their possible significance in breeding programs to enhance yield and adaptability.

The high CV values recorded for single fruit weight (73.16%) and five-fruit weight (64.38%) indicate that these traits are subject to strong environmental and/or genetic influences, and they represent promising targets for selection in breeding programs aimed at improving fruit yield (Table 2). However, these interpretations are based on phenotypic observations, and heritability estimates were not determined in the present study. In contrast, leaflet number exhibited the lowest variability (CV = 12.44%), suggesting that this trait may be under more conservative genetic control and is less responsive to environmental [51,74]. These observations align with the findings of Eshghi and Akhundova [27], who similarly documented differential CV profiles across morphological characters in *Hordeum vulgare* germplasm, and with Maqsood Ahmed *et al*., [26], who reported analogous variability patterns in wild *Rubus idaeus* from Azad Jammu and Kashmir.

Pearson correlation analysis revealed biologically meaningful associations among morphological traits. The strong positive correlation between single fruit weight and five-fruit weight (*r* = 0.98**) confirms the reliability of the five-fruit weight parameter as a yield proxy. The significant correlation between internode length and fruit weight (*r* = 0.60*) suggests that vegetative architecture—specifically node spacing—may influence resource allocation to fruit development. The negative correlation between leaf length and plant height (*r* = −0.55*) may reflect a trade-off between vegetative growth strategies, with taller, more erect genotypes potentially investing proportionally less in foliar area (Fig 3). These correlations are consistent with the concept of pleiotropy in quantitative trait loci and may have practical implications for simultaneous indirect selection of multiple agronomically desirable traits [39,74].

PCA effectively resolved the multidimensional morphological variation into interpretable principal components, collectively explaining 60.20% of total variance (Fig 4). The dominance of fruit weight-related variables in the first two components confirms that reproductive yield traits represent the primary axis of differentiation among the studied landraces. The clear segregation of genotypes (HBS) and (BKK) from the remainder of the panel in the PCA biplot (Fig 4) identifies these accessions as particularly divergent and potentially valuable for use in crosses targeting improved fruit production. These findings are consistent with applications of PCA in other *Rubus* studies [42,75], and with broader applications of multivariate analysis to germplasm characterization in legumes and cereals [37,38,40]. The clustering pattern of (Fig 5) is correlated with PCA results, as the two analyses point to similar clusters of genotypes and reveal the existence of a significant morphological diversity.

SDS-PAGE analysis of seed storage proteins revealed 21 polymorphic bands within the molecular weight range of 14.4–97.4 kDa, confirming substantial biochemical diversity among the fifteen landraces. Shannon’s diversity index values highlighted high polymorphism in markers B-03, B-06, B-08, B-12, and B-21, while several other markers were conserved across all genotypes (H′ = 0.00) (Table 3). These results are consistent with previous applications of SDS-PAGE in diversity assessment of legumes and other species [36,37,43,44,59] and demonstrate that seed protein profiling provides a cost-effective biochemical approach for initial diversity screening in resource-limited settings. Notably, the relatively high CV values for certain markers (up to 93.54%) exceed conventional thresholds for experimental precision, suggesting that some of this variation may reflect genuine allelic polymorphism in storage protein loci rather than purely technical noise. Nevertheless, as emphasized by Chen *et al*., [76], complementing SDS-PAGE with DNA-based molecular markers—such as SSR or SNP genotyping—would substantially strengthen the resolution and accuracy of diversity estimates. The two-way hierarchical cluster dendrogram resolved genotypes into two major lineages, with accessions “(TRG)” and “(SKG)” exhibiting the greatest divergence, consistent with their distinct collection environments [77].

HPLC-UV analysis identified twelve phenolic compounds with significant inter-genotype variation in both presence and quantitative level (Tables 4 and 5). The detection of high quercetin content in genotype (SKG) and elevated epigallocatechin gallate in (TRG) is of particular nutraceutical relevance, given the well-documented antioxidant, anti-inflammatory, and metabolic syndrome-preventive properties of these compounds [61,62]. The complete absence of certain compounds—such as ellagic acid and catechin hydrate—in specific genotypes suggests genotype-specific regulation of secondary metabolite biosynthesis, potentially reflecting differential expression of biosynthetic enzymes in response to local environmental conditions including altitude, soil chemistry, and temperature [24,66,78]. Variation in phenolic profiles among *Rubus* genotypes has similarly been documented in studies from the European Union [78]and other regions [11,23,28], further situating the present findings within the broader literature. This degree of phytochemical heterogeneity among landraces from the same geographic region is noteworthy and represents a significant resource for nutraceutical product development [31,32]. The observed morphological variability, particularly in fruit-related traits, may be associated with differences in phytochemical accumulation, suggesting a potential linkage between phenotypic traits and secondary metabolite profiles.

A further analysis of the HPLC-UV data (Table 5) reveals both the conservation and genotype-specific distribution pattern of phenolic compounds across the seven examined landraces. A number of compounds such as malic acid, gallic acid, vitamin C, chlorogenic acid, epigallocatechin gallate, quercetin, morin, phloroglucinol, and hydroxyl benzoic acid were consistently found in all genotypes indicating their essential presence in the fruit composition of R. fruticosus. On the other hand, the absence of ellagic acid in Binkat Swat, catechin hydrate in Bajaour Pull, and caffeic acid in Shangla indicate differential metabolism among the landraces. (Table 5) indicates the existence or non-existence of phenolic compounds among the landraces, whereas (Table 4) indicates their quantitative changes in form of peak area and percentage.

These compounds are also important due to their functional relevance, as summarized in (Table 6). The most frequently reported compounds with antioxidants and anti-inflammatory activities include gallic acid, quercetin, catechin hydrate, and epigallocatechin gallate, whereas chlorogenic and caffeic acids contribute to antioxidant activity and plant defense [62,65]. The roles are based on previous studies and were not directly evaluated in the present work; however, their presence indicated the potential of these landraces for nutraceutical applications.

The combined morphological, biochemical, and phytochemical evidence presented here substantiates the existence of substantial diversity within the *R. fruticosus* germplasm of the Malakand Division. This diversity constitutes a valuable resource for breeding programs targeting enhanced antioxidant activity, improved fruit quality, and environmental adaptability. The use of altitude- and ecotype-differentiated collection sites likely contributed to the broad diversity captured, and future studies should incorporate more detailed environmental characterization—including soil physicochemical analysis, rainfall data, and temperature profiles—to disentangle the relative contributions of genetic versus environmental factors to observed phenotypic and phytochemical variation. Integration of advanced molecular tools (SSR, SNP, next-generation sequencing) with the biochemical approaches employed here would further enhance the precision of genetic diversity estimates and support informed germplasm conservation and pre-breeding strategies [29,41]. These findings emphasize the need for conservation of indigenous landraces to safeguard genetic diversity and support future breeding efforts.

## Conclusion

The present study establishes a comprehensive baseline characterization of indigenous *Rubus* fruticosus germplasm from Malakand Division, Khyber Pakhtunkhwa — a region critically understudied in the context of *Rubus* diversity. Substantial morphological, biochemical, and phytochemical diversity exists among the fifteen studied landraces, reflecting adaptive potential shaped by the area’s varied ecological conditions. Variation in qualitative and quantitative morphological traits, polymorphism detected through SDS-PAGE seed protein profiling, and genotype-specific differences in phenolic composition collectively confirm that this germplasm constitutes a valuable and largely untapped genetic resource. Genotypes HBS and BKK emerge as promising candidates for breeding programs targeting improved fruit quality, while accessions SKG and TRG stand out for elevated phenolic concentrations, positioning them as strong candidates for functional food and pharmaceutical applications. These findings underscore the urgency of conserving and systematically documenting wild and semi-wild *Rubus* germplasm in mountain agro-ecosystems before ongoing land-use changes erode this diversity irreversibly. Future research incorporating physiological characterization and fine-scale environmental profiling will deepen understanding of local adaptation and translate germplasm diversity into meaningful breeding and conservation outcomes.

## Supporting information

S1 Table Geographic coordinates of *Rubus fruticosus* L. landraces. (DOCX)

## Abbreviations

CV: Coefficient of Variation
FAO: Food and Agriculture Organization
GPS: Global Positioning System
HCA: Hierarchical Cluster Analysis
HPLC-UV: High-Performance Liquid Chromatography with Ultraviolet Detection
IBPGR: International Board for Plant Genetic Resources
IPGRI: International Plant Genetic Resources Institute
kDa: Kilodalton
PCA: Principal Component Analysis
PLS-DA: Partial Least Squares Discriminant Analysis
RAPD: Random Amplification of Polymorphic DNA
SDS-PAGE: Sodium Dodecyl Sulfate–Polyacrylamide Gel Electrophoresis
SNP: Single Nucleotide Polymorphism
SSR: Simple Sequence Repeat
TEMED: Tetramethylethylenediamine
