# Supplementary material for: Morpho-biochemical diversity and phytochemical profiling of Rubus fruticosus L. landraces
Source: PLoS One. 2026 Jul 2;21(7):e0350451. doi: 10.1371/journal.pone.0350451 (PMC13327269; doi:10.1371/journal.pone.0350451)
Supplement: S1 Table — (DOCX) [file pone.0350451.s001.docx]

| Codes | Sites | latitude | longitude |
| --- | --- | --- | --- |
| KT | Kotigram | 34.76068922 | 72.003873 |
| SHZ | Shamozai | 34.68543424 | 72.13217209 |
| BP | Bajaour Pull | 34.72473102 | 71.36554108 |
| SHG | Shangla | 34.86079481 | 72.62150329 |
| BM | Bajaour Mano | 34.80721459 | 71.5263445 |
| BF | Bajaourr palang | 34.73636414 | 71.55194822 |
| TRG | Thana Rotigram | 34.62666518 | 72.08097138 |
| BMK | Bajaour Maya Kalai | 34.73900894 | 71.4159722 |
| SKG | Swat khz gero | 34.93568348 | 72.47024851 |
| BKK | Bajaour Khar Kas | 34.73022582 | 71.5196217 |
| UT | UOM Thana | 34.6630808 | 72.06290614 |
| URT | UOM road Thana | 34.64168303 | 72.06558589 |
| BKS | Binkat Swat | 35.03583654 | 72.48989551 |
| HBS | Hybrid Swat | 35.12609804 | 72.54519524 |
| BSQ | Bajaour Shomlo Qala | 34.88076427 | 71.42222821 |

Supplementary Table 1. Geographic coordinates of Rubus fruticosus L. Landraces
