# Supplementary material for: Morpho-biochemical diversity and phytochemical profiling of Rubus fruticosus L. landraces
Source: PLoS One. 2026 Jul 2;21(7):e0350451. doi: 10.1371/journal.pone.0350451 (PMC13327269; doi:10.1371/journal.pone.0350451)
Supplement: Attachment — Submitted filename: Comments R.docx [file pone.0350451.s002.docx]

## Comments and corrections

1️⃣ Title

You mention “Ethnobotanical Uses” in the title, but:

Ethnobotanical survey methodology is not clearly described in Materials & Methods.

No structured ethnobotanical data (informant number, use value, ICF, RFC etc.) is presented in Results.

Either:

Add proper ethnobotanical methodology and quantitative indices

OR

Remove “Ethnobotanical Uses” from the title.

Also remove study location from title:From Khyber Pakhtunkhwa, Pakistan

2️⃣ Abstract

“Genotyping diversity was assessed by screening of seed proteins”

→ SDS-PAGE is not genotyping, it is biochemical protein profiling.

Also add full abbreviation in abstract and through out MS while using first time Short forms.

HPLC quantification method is unclear (peak area % vs actual concentration).

No mention of sample replication.

PCA variance explanation needs clearer structure.

Correct -

“genetic losses” → “genetic loss”

“highlight their potential” → “highlights their potential”

“with major contributions recorded for plant height” → awkward phrasing

3️⃣ Introduction

1. Repetition

Paragraphs on ecological and cultural significance are repeated (lines 62–74).

Merge them into one concise paragraph.

2. Contradictory Statement

“more recent research has shown that it has the opposite impact on anxiety and cognition”

This contradicts earlier medicinal benefits and is unclear.

3. Weak Research Gap

No integrated study combining morphology + protein + HPLC in KP region.

Lack of conservation data.

Lack of biochemical quantification in wild landraces.

4. Overcitation

Some citations irrelevant (e.g., Vitamin E exercise reference).

Remove unrelated references.

“protein rich and inexpensive with some aspects as supplements” → grammatically incorrect.

“would be responsible for reducing” → “are responsible for reducing”

“characteriszation” → spelling error.

Remove comma spacing errors: “Although,in”

4️⃣ Materials and Methods

4.1 Plant Collection

No GPS coordinates.

No herbarium voucher number.

No altitude data.

“War-affected areas” is irrelevant unless justified scientifically.

Add:

Voucher specimen deposited at which herbarium?

Replication number (n=? per genotype).

Add location cordinates in tabular for as supplementary

4.2 Morphological Study

IBPGR 1982 citation incomplete.

Experimental design missing:

Randomized?

How many plants per genotype?

Field vs natural habitat?

Units inconsistent (feet and cm mixed).

4.3 SDS-PAGE

Molecular weight marker not mentioned.

Band size range not reported (kDa).

Gel documentation method not described.

No reproducibility statement.

4.4 HPLC Method

Gradient program not described (time vs solvent %).

Flow rate missing.

The formula explanation is unclear and incomplete.

This section requires major strengthening for publication in good journal.

4.5 Statistical Analysis

Specify:

Significance level (p<0.05?)

Correlation type (Pearson?)

Data normalization before PCA?

Similarity index used for cluster? (Jaccard? Euclidean?)

5️⃣ Results

Whole result section needs revision

4.1 Morphological Traits, describe

Use consistent decimal places.

4.3 PCA
- “Overall, PCA revealed that fruit yield-related traits and vegetative growth parameters…”

5.3 Cluster Analysis

“genotype were divided”

“linages”

“subcluster”

“was found the most diverse”

Distance measure not specified.

Explain dendrogram cut-off scientifically.

4.6 SDS-PAGE Results

🔹 Weak Interpretation

You mention 21 bands but:

Molecular weight range missing.

No gel image quality discussion.

5.5 HPLC Results

No statistical comparison between genotypes.

No ANOVA results shown.

“These compounds are known for their antioxidants” → “antioxidant properties”

Elaborate result section morphological, biochemical and multivariate analysis

6️⃣ Discussion

1. Discussion repeats results instead of interpreting deeply.
2. No ecological explanation of variation.
3. Overstatement of breeding potential without heritability data.
4. Weak integration between morphology and biochemistry.

🔹 Improve By Adding:

Possible role of altitude, soil type.

Environmental vs genetic influence.

Conservation implications.

Comparison with other Rubus studies globally.

7️⃣ Conclusion

Too descriptive.

Repetition of results.

“physiological variation” not studied.

8️⃣ References

Formatting inconsistent.

Journal names inconsistent.

Some incomplete citations.

Add DOI in all ref

Add some latest reference

Duplicate Begna citation.

FAO citation incomplete.
