# Supplementary material for: Morpho-biochemical diversity and phytochemical profiling of Rubus fruticosus L. landraces
Source: PLoS One. 2026 Jul 2;21(7):e0350451. doi: 10.1371/journal.pone.0350451 (PMC13327269; doi:10.1371/journal.pone.0350451)
Supplement: Attachment — Submitted filename: Response_to_reviewers.docx [file pone.0350451.s004.docx]

PONE-D-25-56551

Biochemical Profiling and Ethnobotanical Uses of Rubus fruticosus L. in Khyber Pakhtunkhwa, Pakistan

PLOS One

Dear Dr. Khan,

Thank you for submitting your manuscript to PLOS ONE. After careful consideration, we feel that it has merit but does not fully meet PLOS ONE’s publication criteria as it currently stands. Therefore, we invite you to submit a revised version of the manuscript that addresses the points raised during the review process.

Please submit your revised manuscript by Apr 19 2026 11:59PM.

1. Please ensure that your manuscript meets PLOS ONE's style requirements, including those for file naming. The PLOS ONE style templates can be found at

<https://journals.plos.org/plosone/s/file?id=wjVg/PLOSOne_formatting_sample_main_body.pdf> and

<https://journals.plos.org/plosone/s/file?id=ba62/PLOSOne_formatting_sample_title_authors_affiliations.pdf>

2. In your Methods section, please provide additional information regarding the permits you obtained for the work. Please ensure you have included the full name of the authority that approved the field site access and, if no permits were required, a brief statement explaining why.

**Response:** We thank the reviewer for this suggestion. We have revised the manuscript accordingly and included the full name of the approving authority along with a brief statement explaining that” The study was conducted in compliance with applicable institutional, national, and international guidelines for field collection of plant material. Ethical approval was granted by the Institutional Ethics Committee of the University of Malakand. Collection of plant material from the study sites did not require special government permits, as the sampled areas constitute non-protected forest zones in the Malakand Division; however, all collections adhered to local land-use regulations” on pager no. 04, line no. 146 to 153.

3. Please include your tables as part of your main manuscript and remove the individual files. Please note that supplementary tables (should remain/ be uploaded) as separate "supporting information" files.

**Response:** We thank the reviewer for this instruction. We have revised the manuscript accordingly by incorporating all main tables directly into the body of the manuscript at their appropriate locations. Supplementary tables have been retained as separate "Supporting Information" files and uploaded accordingly. We hope these revisions meet the journal's formatting requirements.

4. We note that your Data Availability Statement is currently as follows: All relevant data are contained within the manuscript and its Supporting Information files.

Please confirm at this time whether or not your submission contains all raw data required to replicate the results of your study. Authors must share the “minimal data set” for their submission. PLOS defines the minimal data set to consist of the data required to replicate all study findings reported in the article, as well as related metadata and methods (<https://journals.plos.org/plosone/s/data-availability#loc-minimal-data-set-definition>).

For example, authors should submit the following data:

- The values behind the means, standard deviations and other measures reported;
- The values used to build graphs;
- The points extracted from images for analysis.

Authors do not need to submit their entire data set if only a portion of the data was used in the reported study.

If your submission does not contain these data, please either upload them as Supporting Information files or deposit them to a stable, public repository and provide us with the relevant URLs, DOIs, or accession numbers. For a list of recommended repositories, please see <https://journals.plos.org/plosone/s/recommended-repositories>.

If there are ethical or legal restrictions on sharing a de-identified data set, please explain them in detail (e.g., data contain potentially sensitive information, data are owned by a third-party organization, etc.) and who has imposed them (e.g., an ethics committee). Please also provide contact information for a data access committee, ethics committee, or other institutional body to which data requests may be sent. If data are owned by a third party, please indicate how others may request data access.

**Response:** Thank you for drawing our attention to the data availability requirement. We confirm that our submission contains all raw data necessary to replicate the study findings. The values underlying all reported means, standard deviations, and statistical outputs — including those used to construct figures and tables — are provided within the manuscript text and the accompanying Supporting Information files. No additional data were generated or used beyond what is already included. We are therefore satisfied that the current Data Availability Statement accurately reflects the status of our submission.

5. If the reviewer comments include a recommendation to cite specific previously published works, please review and evaluate these publications to determine whether they are relevant and should be cited. There is no requirement to cite these works unless the editor has indicated otherwise. 

**Response:** We have carefully reviewed the recommended publications and evaluated their relevance to our study. Where appropriate, relevant citations have been incorporated into the revised manuscript to strengthen the context and discussion. Publications that were not directly aligned with the scope and objectives of our work have not been included.


Reviewers' comments:

Reviewer's Responses to Questions

**Comments to the Author**

1. Is the manuscript technically sound, and do the data support the conclusions?

The manuscript must describe a technically sound piece of scientific research with data that supports the conclusions. Experiments must have been conducted rigorously, with appropriate controls, replication, and sample sizes. The conclusions must be drawn appropriately based on the data presented.

Reviewer #1: Partly

Reviewer #2: Yes

Reviewer #3: Partly

**Response:**

We thank Reviewers 1 and 3 for their partial assessments and Reviewer 2 for the positive evaluation. We have carefully revisited the manuscript in light of the concerns raised and have strengthened the relevant sections to ensure that the conclusions are more clearly and rigorously grounded in the data presented.

2. Has the statistical analysis been performed appropriately and rigorously?

Reviewer #1: Yes

Reviewer #2: Yes

Reviewer #3: Yes

**Response:** We are pleased that all three reviewers considered the statistical analysis appropriate and rigorous.

3. Have the authors made all data underlying the findings in their manuscript fully available?

The [PLOS Data policy](http://track.editorialmanager.com/CL0/http:%2F%2Fwww.plosone.org%2Fstatic%2Fpolicies.action%23sharing/1/010f019cbe75345b-8765400a-43c6-4ef7-91c5-48a5727c2da1-000000/5hxMu21XvJ5Pdl3EVp_FnKC0s-7fRL4Sx74BFToLEuY=253) requires authors to make all data underlying the findings described in their manuscript fully available without restriction, with rare exception (please refer to the Data Availability Statement in the manuscript PDF file). The data should be provided as part of the manuscript or its supporting information, or deposited to a public repository. For example, in addition to summary statistics, the data points behind means, medians and variance measures should be available. If there are restrictions on publicly sharing data—e.g. participant privacy or use of data from a third party—those must be specified.

Reviewer #1: Yes

Reviewer #2: Yes

Reviewer #3: Yes

**Response:** We are grateful that all reviewers confirmed the data underlying the findings are fully available. Our Data Availability Statement has been reviewed and remains accurate.

4. Is the manuscript presented in an intelligible fashion and written in standard English?

PLOS ONE does not copyedit accepted manuscripts, so the language in submitted articles must be clear, correct, and unambiguous. Any typographical or grammatical errors should be corrected at revision, so please note any specific errors here.

Reviewer #1: Yes

Reviewer #2: No

Reviewer #3: No

**Response:**

We acknowledge the concerns raised by Reviewers 2 and 3 regarding language clarity. The manuscript has been carefully re-read and revised throughout to correct typographical and grammatical errors and to improve overall clarity and readability. We are confident the revised version meets the standard expected for publication in PLOS ONE.

5. Review Comments to the Author

Please use the space provided to explain your answers to the questions above. You may also include additional comments for the author, including concerns about dual publication, research ethics, or publication ethics. (Please upload your review as an attachment if it exceeds 20,000 characters)

**Reviewer #1: major revision**
**Reasons.**
As the author mentions in the results section, with the heading (Genetic Diversity and Shannon Diversity Index) and table (Table 5. Percent Coefficient of variation and Shannon diversity index in Rubus fruiticosus seed protein profile using SDS-PAGE), the CV values exceed (35.36-93.54%) the general acceptable limits of research experiments.

**Response:** We appreciate the reviewer’s careful evaluation of our results. However, it is important to clarify that the coefficient of variation (CV) values reported in table 5 now table 3 represent biological variability among Rubus fruticosus genotypes in seed storage protein profiles, rather than variability arising from experimental or methodological error.

In studies focused on genetic diversity, germplasm characterization, and biochemical marker analysis (e.g., SDS-PAGE protein profiling), relatively high CV values are expected because the purpose of the analysis is to capture polymorphism and heterogeneity among genotypes. Unlike controlled agronomic experiments where CV is commonly used as an indicator of experimental precision, in diversity studies CV reflects the extent of phenotypic or biochemical variation present within the population.

The CV values observed in our study (35.36–93.54%) correspond to differences in presence/absence and intensity of protein bands among the analyzed accessions, which is consistent with the inherent genetic heterogeneity of Rubus fruticosus populations.

Furthermore, the Shannon Diversity Index values presented in Table 5 now table 3 corroborate the presence of considerable genetic diversity, supporting the interpretation that the observed variation is biologically meaningful and relevant to the objectives of the study.

Therefore, the reported CV values should not be interpreted as exceeding acceptable experimental limits but rather as reflecting the natural genetic diversity captured by the SDS-PAGE protein profiling approach. To avoid potential misunderstanding, we have revised the manuscript to clarify that the CV values represent genotypic variability rather than experimental imprecision.

**2. In abstract, authors must highlight the statistical research design used for quantitative traits.**

**Response:** We thank the reviewer for this suggestion. The abstract has been revised to explicitly state that the experiment was conducted using a Randomized Complete Block Design (RCBD).

3. Avoid scientific names in keywords; if added, then they should be italic.

**Response:** We thank the reviewer for this observation. The keywords have been revised accordingly. Scientific names that were not essential as keywords have been removed. Where a scientific name has been retained due to its relevance, it has been italicized as per standard convention.

4. In the introduction, the authors used 10-27 years or older in-text citations, which should be recent ones. The author should read the manuscript carefully; some sentences require correction to make them more accurate and scientifically sound.

**Response:** We thank the reviewer for this valid observation. The introduction has been thoroughly revised to replace outdated citations with more recent and relevant literature, ensuring that the references better reflect the current state of knowledge in the field. Additionally, the manuscript has been carefully re-read, and sentences that were identified as unclear or insufficiently precise have been revised to improve scientific accuracy and readability throughout.

5. In the material and methods section
i. Enlist the studies' landraces' names, if possible, then also add origins.
ii. Also mentioned clearly, for an ethnobotanical study, any survey conducted or just literature cited data added, as mentioned in Table 1.
iii. Mention the exact date of the experiment start and end date/seasons, along with the research design.

**Response:** The studied landraces names were represented in Figure 2 and origin were included as well. The ethnobotanical information was excluded from the study and the study date and year were mentioned as suggested.

6. In the results section, Table 6 shows the phenolic identifications of 7 landraces, whereas in the materials and methods, 15 landraces are mentioned, which are studied. Correlation analysis should be represented in a figure instead of a table. Similarly, in the PCA, no need to add the table and eigenvalue values; just add the PCA biplot. In Figure 1, there is an overlap of values.

**Response:** Thank you for highlighting this point. Although fifteen landraces were initially included in the study (as described in the Materials and Methods section), only seven landraces are presented in Table 6 for phenolic identification. This is because detailed phenolic profiling was conducted on a subset of representative landraces selected based on their variability and significant differences observed in preliminary analyses (e.g., total phenolic content and antioxidant activity). The remaining landraces were excluded from this specific analysis due to limited variation and/or lower relevance to the objective of detailed compound identification.

Correlation analysis was represented as Figure 3 and the PCA results table showing eigenvalues has been removed, and only the PCA biplot has been retained in the revised manuscript as figure 4.

7. In the discussion part, the current manuscript is supported by a study with CV values is 12.44, which is acceptable in research experiments, while in the present study, the CV is above the acceptable limit, indicating that the collected data is not statistically significant. Add the latest and a few other studies to support the discussion part.

**Response:** Thank you for this important observation. We acknowledge that the coefficient of variation (CV) reported in the present study is higher than that cited in some previous studies (e.g., CV = 12.44). However, CV values are highly dependent on the type of experiment, biological material, and environmental variability, and therefore cannot be interpreted using a single universal threshold.

8. In the conclusion section, levels of physical are mentioned. In the study, no physical traits are studied; morphological and biochemical traits are studied.

**Response:** Thank you for your careful observation. We agree that the term “physical traits” was inaccurately used in the conclusion section. The present study specifically focuses on morphological and biochemical traits, and no physical traits were evaluated.

Accordingly, the conclusion has been revised to replace “physical traits” with “morphological and biochemical traits” to ensure consistency and accuracy with the study design and reported results

9. Need to set referencing according journal format.

**Response:** Thank you for your comment. The references have now been carefully revised and formatted according to the journal’s guidelines. All in-text citations and reference list entries have been checked for consistency, completeness, and accuracy in line with the required style.

**Reviewer #2:** Kindly refer the suggestions in attachment for the manuscript Biochemical Profiling and Ethnobotanical Uses of Rubus fruticosus L. in Khyber Pakhtunkhwa, Pakistan.

The MS needs major revision before considering it.

## Reviwer 2. Comments and corrections

**1️. Title**

You mention “Ethnobotanical Uses” in the title, but: Ethnobotanical survey methodology is not clearly described in Materials & Methods.

No structured ethnobotanical data (informant number, use value, ICF, RFC etc.) is presented in Results.

Either: Add proper ethnobotanical methodology and quantitative indices OR Remove “Ethnobotanical Uses” from the title. Also remove study location from title:From Khyber Pakhtunkhwa, Pakistan

**Response:**

We thank the reviewer for this valuable suggestion. We agree that the ethnobotanical component was not supported by detailed field-based methodology or quantitative ethnobotanical indices such as informant number, use value (UV), informant consensus factor (ICF), or relative frequency of citation (RFC). To avoid any potential misunderstanding regarding the scope of the study, the ethnobotanical component has been removed from the revised manuscript.

Accordingly, the title has been revised to remove “Ethnobotanical Uses” and the study location, ensuring that it accurately reflects the core focus of the research. The revised title is: “Morpho-Biochemical Diversity and Phytochemical Profiling of Rubus fruticosus L. Landraces.”

In addition, the objectives and manuscript structure have been updated to clearly emphasize the evaluation of morphological traits, seed protein polymorphism (SDS-PAGE), and phytochemical composition (HPLC-UV) for the characterization of R. fruticosus landraces. These revisions have improved the clarity and consistency between the title, objectives, methodology, and results of the manuscript.

**2️. Abstract**

“Genotyping diversity was assessed by screening of seed proteins”

→ SDS-PAGE is not genotyping, it is biochemical protein profiling.

Also add full abbreviation in abstract and through out MS while using first time Short forms.

HPLC quantification method is unclear (peak area % vs actual concentration).

No mention of sample replication.

PCA variance explanation needs clearer structure.

Correct -

“genetic losses” → “genetic loss”

“highlight their potential” → “highlights their potential”

“with major contributions recorded for plant height” → awkward phrasing

**Response:**

We thank the reviewer for the careful evaluation of the manuscript and for these helpful suggestions. The manuscript has been revised accordingly as described below:

Clarification of SDS-PAGE terminology

We agree with the reviewer that SDS-PAGE analysis represents biochemical seed protein profiling rather than genotyping. Therefore, the statement has been corrected throughout the manuscript. The revised text now reads:

“Biochemical diversity was assessed through sodium dodecyl sulfate–polyacrylamide gel electrophoresis (SDS-PAGE) based seed protein profiling.”

Abbreviations

All abbreviations have now been defined at their first appearance in the Abstract and throughout the manuscript, including SDS-PAGE (Sodium Dodecyl Sulfate–Polyacrylamide Gel Electrophoresis), HPLC (High Performance Liquid Chromatography), and PCA (Principal Component Analysis).

Clarification of HPLC quantification method

The description of the HPLC method has been revised to clearly indicate the quantification procedure and whether results are expressed as peak area percentage or calculated concentrations using calibration curves. Additional details regarding the analytical procedure have been added in the Materials and Methods section.

Sample replication

Information regarding biological and/or analytical replication has now been added to the Materials and Methods section to clarify the experimental design and ensure reproducibility of the results.

PCA explanation

The Principal Component Analysis (PCA) description has been revised and restructured to more clearly present the variance explained by the principal components and the contribution of individual traits.

Minor language corrections

The suggested corrections have been implemented throughout the manuscript, including:

“genetic losses” → “genetic loss”

“highlight their potential” → “highlights their potential”

The phrase “with major contributions recorded for plant height” has been revised to improve clarity and readability.

3️⃣ Introduction

1. Repetition

Paragraphs on ecological and cultural significance are repeated (lines 62–74).

Merge them into one concise paragraph.

2. Contradictory Statement

“more recent research has shown that it has the opposite impact on anxiety and cognition”

This contradicts earlier medicinal benefits and is unclear.

3. Weak Research Gap

No integrated study combining morphology + protein + HPLC in KP region.

Lack of conservation data.

Lack of biochemical quantification in wild landraces.

4. Overcitation

Some citations irrelevant (e.g., Vitamin E exercise reference).

Remove unrelated references.

“protein rich and inexpensive with some aspects as supplements” → grammatically incorrect.

“would be responsible for reducing” → “are responsible for reducing”

“characteriszation” → spelling error.

Remove comma spacing errors: “Although,in”

**Response:**
The Introduction section has been revised to remove repetition, improve clarity, and correct grammatical and typographical issues. The contradictory statement has been deleted. The research gap has been strengthened by clearly highlighting the lack of integrated morphological, biochemical (SDS-PAGE), and phytochemical (HPLC) studies in *Rubus fruticosus* landraces from the region, along with limited quantitative biochemical data. Irrelevant citations were reviewed and corrected where necessary. These revisions have improved the clarity and scientific focus of the Introduction.

4️⃣ Materials and Methods

4.1 Plant Collection

No GPS coordinates.

No herbarium voucher number.

No altitude data.

“War-affected areas” is irrelevant unless justified scientifically.

Add:

Voucher specimen deposited at which herbarium?

Replication number (n=? per genotype).

Add location cordinates in tabular for as supplementary

4.2 Morphological Study

IBPGR 1982 citation incomplete.

Experimental design missing:

Randomized?

How many plants per genotype?

Field vs natural habitat?

Units inconsistent (feet and cm mixed).

4.3 SDS-PAGE

Molecular weight marker not mentioned.

Band size range not reported (kDa).

Gel documentation method not described.

No reproducibility statement.

4.4 HPLC Method

Gradient program not described (time vs solvent %).

Flow rate missing.

The formula explanation is unclear and incomplete.

This section requires major strengthening for publication in good journal.

4.5 Statistical Analysis

Specify:

Significance level (p<0.05?)

Correlation type (Pearson?)

Data normalization before PCA?

Similarity index used for cluster? (Jaccard? Euclidean?)

Response

In the revised manuscript, we have comprehensively addressed all concerns. Specifically, we have added GPS coordinates, altitude data, herbarium voucher information, and clarified replication details for plant collection. The experimental design for morphological characterization has been clearly described, including the randomized complete block design (RCBD), number of plants per genotype, and field conditions, with consistent use of measurement units. The IBPGR reference has been corrected. For SDS-PAGE analysis, we have included the molecular weight marker range, band size information, gel documentation method, and reproducibility details. The HPLC section has been strengthened by adding the complete gradient program, flow rate, and clarifying the quantification formula. In the statistical analysis section, we have specified the significance level, correlation method (Pearson), data standardization prior to PCA, and the use of Euclidean distance for cluster analysis. We believe these revisions have significantly improved the clarity, rigor, and reproducibility of the methodology.

5️⃣ Results

Whole result section needs revision

4.1 Morphological Traits, describe

Use consistent decimal places.

4.3 PCA
- “Overall, PCA revealed that fruit yield-related traits and vegetative growth parameters…”

5.3 Cluster Analysis

“genotype were divided”

“linages”

“subcluster”

“was found the most diverse”

Distance measure not specified.

Explain dendrogram cut-off scientifically.

4.6 SDS-PAGE Results

🔹 Weak Interpretation

You mention 21 bands but:

Molecular weight range missing.

No gel image quality discussion.

5.5 HPLC Results

No statistical comparison between genotypes.

No ANOVA results shown.

“These compounds are known for their antioxidants” → “antioxidant properties”

Elaborate result section morphological, biochemical and multivariate analysis

**Response:**
The entire Results section has been comprehensively revised to improve clarity, scientific flow, and consistency. Redundant statements were removed, and the structure was reorganized into clearly defined subsections covering morphological, multivariate, biochemical, and phytochemical analyses.

The morphological traits section has been expanded and clarified. Both qualitative and quantitative trait descriptions were improved, and inconsistencies in genotype reference and terminology were corrected for accuracy and clarity.

All quantitative morphological data were standardized to consistent decimal places across Tables 2 and related text to ensure uniform reporting and improved readability.

The PCA section has been revised with a clearer interpretation of principal components and trait contributions. An explicit summary statement was added to highlight that fruit yield-related and vegetative growth traits were the major contributors to morphological variation among genotypes.

The hierarchical cluster analysis section was revised as follows:

- Corrected grammatical errors (“genotype were divided” → “genotypes were divided”)
- Corrected terminology (“linages” → “lineages”, “subcluster” → “sub-cluster”)
- Clarified identification of most diverse genotypes
- Specified the distance metric (Euclidean distance using Ward’s method)
- Provided a scientific justification for dendrogram cut-off selection based on similarity/dissimilarity structure

The SDS-PAGE results section was strengthened by:

- Adding the molecular weight range (14.4–97.4 kDa)
- Improving interpretation of banding patterns
- Clarifying polymorphism across genotypes
- Enhancing discussion of genetic diversity indicated by protein profiles

The HPLC-UV results section was revised to clarify that the analysis was descriptive in nature due to targeted metabolite profiling. A statement was added explaining that no ANOVA or inferential statistical comparison was performed, and results are presented based on comparative abundance and presence/absence patterns across genotypes.

The sentence was corrected and refined to scientifically appropriate terminology. The phrase was revised to refer to “antioxidant properties” instead of informal wording, ensuring technical accuracy.

The Results section has been fully reorganized and strengthened by integrating morphological, biochemical, multivariate, and phytochemical analyses in a coherent structure. Interpretation has been improved without overlapping with Materials and Methods, ensuring alignment with standard scientific reporting practices.

**6️⃣ Discussion**

1. Discussion repeats results instead of interpreting deeply.
2. No ecological explanation of variation.
3. Overstatement of breeding potential without heritability data.
4. Weak integration between morphology and biochemistry.

🔹 Improve By Adding:

Possible role of altitude, soil type.

Environmental vs genetic influence.

Conservation implications.

Comparison with other Rubus studies globally.

**Response**

We thank the reviewer for the constructive feedback. The Discussion section has been revised accordingly.

The section has been revised to reduce repetition of results and to provide deeper interpretation with biological explanations of the observed variation.

Explanations related to altitude, soil type, moisture, and temperature have been incorporated to clarify environmental influences.

Statements regarding breeding potential have been moderated, and a limitation noting the absence of heritability estimates has been added.

A clearer link between morphological traits (particularly fruit-related traits) and phytochemical variation has been included to improve integration between datasets.

The roles of genetic variation and phenotypic plasticity have been clearly distinguished to explain environmental and genetic influences.

A statement emphasizing the importance of conserving indigenous landraces has been added in the conclusion section.

Relevant comparisons with previous Rubus studies from different regions have been incorporated to contextualize the findings within global literature.

These revisions have improved the clarity, interpretation, and scientific strength of the Discussion section.

7️⃣ Conclusion

Too descriptive.

Repetition of results.

“physiological variation” not studied.

**Response**

The Conclusion section has been revised to remove excessive descriptive detail and repeated results, and to improve synthesis and interpretation. Redundant reporting of numerical values and trait-level summaries has been minimized, and the content has been rewritten in a more concise, conclusion-oriented format. Statements that previously resembled Results have been reframed to reflect overall interpretation and study implications. In addition, a clear limitation has been added indicating that physiological traits and detailed environmental factors were not assessed in the present study, addressing concerns regarding overgeneralization and methodological scope.

8️⃣ References

Formatting inconsistent.

Journal names inconsistent.

Some incomplete citations.

Add DOI in all ref

Add some latest reference

Duplicate Begna citation.

FAO citation incomplete.

**Response:**

Thank you for your detailed comments regarding the reference section. We have carefully revised the entire reference list to ensure compliance with the journal’s formatting requirements.

Specifically, we have:

Standardized the formatting of all references for consistency

Corrected inconsistencies in journal names (ensuring uniform style throughout)

Completed all incomplete citations with missing bibliographic details

Added DOIs to all references where available

Included recent and relevant updated references to strengthen the manuscript

Removed the duplicate Begna citation

Corrected and completed the FAO citation with full details

These revisions have improved the accuracy, consistency, and scholarly quality of the reference section.

Reviewer #3: Please address the following points
1. The article combines many studies such as ethnobotanical documentation, morphological characterization, seed protein profiling and phytochemical analysis, however, combining all these studies are insufficiently justified. The objective of the study is quite broad and divided into many parts, which seems quite difficult to understand the main theme and hypothesis of the study. I will suggest that the authors should clearly define wether the study primarily address conservation, genetic diversity, biochemical characterization, or ethnobotanical analysis, and restructure the manuscript accordingly.

**Response:**

We sincerely thank the reviewer for this constructive suggestion. In response, the manuscript has been carefully revised to clarify the central theme and research objectives.

First, the ethnobotanical component has been removed from the manuscript to avoid excessive breadth and to maintain a more focused scientific framework. The revised study now specifically concentrates on the morphological, biochemical, and phytochemical diversity of Rubus fruticosus landraces.

Second, the main objective of the study has been refined and clearly stated in the Introduction. The primary aim is to characterize the diversity among R. fruticosus landraces using complementary approaches, including morphological traits, SDS-PAGE–based seed protein profiling, and HPLC-UV phytochemical analysis. These methods were intentionally integrated to provide a comprehensive evaluation of diversity at phenotypic, protein, and metabolite levels, which is essential for effective germplasm characterization and identification of promising genotypes for future breeding and utilization.

Third, the manuscript has been restructured to improve clarity and coherence, ensuring that all sections consistently reflect the unified objective of assessing morpho-biochemical diversity and phytochemical composition of the studied landraces.

We believe that these revisions significantly improve the focus of the manuscript and clearly establish the study’s primary emphasis on diversity characterization of Rubus fruticosus germplasm, while also highlighting its relevance for genetic resource evaluation and potential breeding applications.

2. IBPGR descriptors, SDS-PAGE seed profiling and HPLC phytochemical analysis are well established methodologies and mostly used in diversity studies. What is the novelty or new biological insights beyond descriptive variability assessment. Is it clear in the current form that the novelty relative to existing target plant diversity studies especially from south Asia, should be clearly presented.

**Response:**

We thank the reviewer for this insightful comment and for highlighting the importance of clearly emphasizing the novelty and biological relevance of the study. We agree that the methodologies employed in this work—IBPGR morphological descriptors, SDS-PAGE seed protein profiling, and HPLC-based phytochemical analysis—are well-established approaches for diversity studies. However, the novelty of the present research lies in the integrated application of these complementary approaches to characterize Rubus fruticosus landraces from the Malakand Division of Khyber Pakhtunkhwa, Pakistan, a region where the genetic and biochemical diversity of this species has been largely unexplored.

Unlike previous studies that typically focus on single-level characterization (either morphological, molecular, or phytochemical), our study provides a multi-level assessment of diversity by combining phenotypic traits, seed protein polymorphism, and quantitative phytochemical profiling within the same germplasm set. This integrated approach allows for a more comprehensive understanding of variation across morphological, biochemical, and metabolite levels, which is valuable for identifying promising genotypes with both agronomic and nutraceutical potential.

Furthermore, the study generates baseline data on the phytochemical composition (phenolic acids, anthocyanins, and vitamins) and protein band polymorphism of R. fruticosus landraces from South Asia, a region where systematic characterization of this species remains limited. The findings provide new biological insights into the extent of diversity present in local germplasm and highlight genotypes with superior phytochemical profiles, which may be valuable for future breeding programs, conservation strategies, and the development of functional foods or nutraceutical products.

To address the reviewer’s concern, the novelty and scientific contribution of the study have now been more clearly emphasized in the Introduction and Discussion sections of the revised manuscript.

3. As the title show the ethnobotanical uses, but the provided section do not carefully describe field based ethnobotanical data collection, informant demographics, survey methodology or validation of traditional uses. In the current form the title of the work is misleading.

**Response:**

We sincerely thank the reviewer for this important observation. We agree that the ethnobotanical component was not sufficiently supported by detailed field-based methodology, including informant demographics, survey design, and validation procedures. To address this concern and to avoid any potential misinterpretation of the study scope, the ethnobotanical section has been completely removed from the revised manuscript.

Accordingly, the title, objectives, and manuscript structure have been revised to reflect the actual focus of the study, which is now centered on the morphological, biochemical, and phytochemical characterization of Rubus fruticosus landraces. The revised title is: “Morpho-Biochemical Diversity and Phytochemical Profiling of Rubus fruticosus L. Landraces.” These revisions ensure that the title accurately represents the scope and methodology of the study. We believe that the manuscript is now more focused and consistent with the objectives and analyses presented.

4. Who identified these plants must be mentioned in material and methods

**Response:** The plant materials used in this study were taxonomically identified by Dr. Mohammad Nisar, a taxonomic botanist at the Department of Botany, University of Malakand, using standard and established regional flora identification keys. The identity of each accession was confirmed as Rubus fruticosus L.

5. The results presented are mainly descriptive, reporting ranges, percentages and variability without provide sufficient biological interpretation. Most of the statements are already presented in the tables and figures. The authors should focus on highlighting key patterns, relationships and meaningful data rather than repeating data.

**Response:** Thank you for this valuable comment. The Results section has been thoroughly revised to reduce redundancy and improve clarity. We have removed repetitive descriptions and now focus on highlighting the key patterns, trends, and relationships observed in the data rather than restating numerical values.

In addition, we have strengthened the biological interpretation of the results by linking observed variations in morphological and biochemical traits to possible genetic and environmental influences, where appropriate.

6. There is no detailed information regarding the environmental condition, replication within sites, that can influence morphological traits.

**Response:** In the revised manuscript, we have now added a detailed description of the study environment, including key environmental conditions of the sampling sites. We have also clarified the experimental design and replication strategy within sites, to better explain how morphological data were collected and how environmental variability was accounted for.

These additions improve the transparency and robustness of the methodology and help better interpret the observed variation in morphological traits.

7. In my places the species names are not italic, also the pattern is not uniform

**Response:** The manuscript has now been thoroughly revised to ensure that all species names are formatted in italics throughout the text, tables, and figures, in accordance with standard taxonomic and journal formatting guidelines. Additionally, consistency in species naming has been ensured across the entire manuscript

8. The whole manuscript should be check for typo errors, also some of the sentences need to be organized well

**Response:** The entire manuscript has been carefully reviewed to correct typographical errors and improve overall language quality. In addition, several sentences have been reorganized and refined to enhance clarity, coherence, and scientific readability.

We have also ensured consistency in terminology and improved the flow of the text throughout the manuscript**.**

6. PLOS authors have the option to publish the peer review history of their article ([what does this mean?](https://track.editorialmanager.com/CL0/https:%2F%2Fjournals.plos.org%2Fplosone%2Fs%2Feditorial-and-peer-review-process%23loc-peer-review-history/1/010f019cbe75345b-8765400a-43c6-4ef7-91c5-48a5727c2da1-000000/CzmMALVPMxK3biSzMJZdTeEE3--rBO9Pu5Pa17LFHHI=253)). If published, this will include your full peer review and any attached files.

If you choose “no”, your identity will remain anonymous but your review may still be made public.

**Do you want your identity to be public for this peer review?** For information about this choice, including consent withdrawal, please see our [Privacy Policy](https://track.editorialmanager.com/CL0/https:%2F%2Fwww.plos.org%2Fprivacy-policy/1/010f019cbe75345b-8765400a-43c6-4ef7-91c5-48a5727c2da1-000000/Yw4kNJUrwUt_BEC3a3vjc-K8-AM6UahxGGmJ4tISBww=253).

Reviewer #1: **Yes:** Imran Ul Haq

Reviewer #2: No

Reviewer #3: No
