# Supplementary material for: Morpho-biochemical diversity and phytochemical profiling of Rubus fruticosus L. landraces
Source: PLoS One. 2026 Jul 2;21(7):e0350451. doi: 10.1371/journal.pone.0350451 (PMC13327269; doi:10.1371/journal.pone.0350451)
Supplement: Peer review history

# Peer review: Decision Letter 0

**DOI:** 10.1371/journal.pone.0350451.r001

**Authors:** Alia Ahmed

5 Mar 2026

PONE-D-25-56551

Biochemical Profiling and Ethnobotanical Uses of Rubus fruticosus L. in Khyber Pakhtunkhwa, Pakistan

PLOS One

Dear Dr. Khan,

Thank you for submitting your manuscript to PLOS ONE. After careful consideration, we feel that it has merit but does not fully meet PLOS ONE’s publication criteria as it currently stands. Therefore, we invite you to submit a revised version of the manuscript that addresses the points raised during the review process.

Please submit your revised manuscript by Apr 19 2026 11:59PM. If you will need more time than this to complete your revisions, please reply to this message or contact the journal office at plosone@plos.org. When you're ready to submit your revision, log on to https://www.editorialmanager.com/pone/ and select the 'Submissions Needing Revision' folder to locate your manuscript file.

Please include the following items when submitting your revised manuscript:

- A letter that responds to each point raised by the academic editor and reviewer(s). You should upload this letter as a separate file labeled 'Response to Reviewers'.
- A marked-up copy of your manuscript that highlights changes made to the original version. You should upload this as a separate file labeled 'Revised Manuscript with Track Changes'.
- An unmarked version of your revised paper without tracked changes. You should upload this as a separate file labeled 'Manuscript'.

If you would like to make changes to your financial disclosure, please include your updated statement in your cover letter. Guidelines for resubmitting your figure files are available below the reviewer comments at the end of this letter.

If applicable, we recommend that you deposit your laboratory protocols in protocols.io to enhance the reproducibility of your results. Protocols.io assigns your protocol its own identifier (DOI) so that it can be cited independently in the future. For instructions see: https://journals.plos.org/plosone/s/submission-guidelines#loc-laboratory-protocols. Additionally, PLOS ONE offers an option for publishing peer-reviewed Lab Protocol articles, which describe protocols hosted on protocols.io. Read more information on sharing protocols at https://plos.org/protocols?utm_medium=editorial-email&utm_source=authorletters&utm_campaign=protocols.

We look forward to receiving your revised manuscript.

Kind regards,

Alia Ahmed

Academic Editor

PLOS One

Journal Requirements:

When submitting your revision, we need you to address these additional requirements.

1. Please ensure that your manuscript meets PLOS ONE's style requirements, including those for file naming. The PLOS ONE style templates can be found at

https://journals.plos.org/plosone/s/file?id=wjVg/PLOSOne_formatting_sample_main_body.pdf and

https://journals.plos.org/plosone/s/file?id=ba62/PLOSOne_formatting_sample_title_authors_affiliations.pdf

2. In your Methods section, please provide additional information regarding the permits you obtained for the work. Please ensure you have included the full name of the authority that approved the field site access and, if no permits were required, a brief statement explaining why.

3. Please include your tables as part of your main manuscript and remove the individual files. Please note that supplementary tables (should remain/ be uploaded) as separate "supporting information" files.

4. We note that your Data Availability Statement is currently as follows: All relevant data are contained within the manuscript and its Supporting Information files.

Please confirm at this time whether or not your submission contains all raw data required to replicate the results of your study. Authors must share the “minimal data set” for their submission. PLOS defines the minimal data set to consist of the data required to replicate all study findings reported in the article, as well as related metadata and methods (https://journals.plos.org/plosone/s/data-availability#loc-minimal-data-set-definition).

For example, authors should submit the following data:

- The values behind the means, standard deviations and other measures reported;

- The values used to build graphs;

- The points extracted from images for analysis.

Authors do not need to submit their entire data set if only a portion of the data was used in the reported study.

If your submission does not contain these data, please either upload them as Supporting Information files or deposit them to a stable, public repository and provide us with the relevant URLs, DOIs, or accession numbers. For a list of recommended repositories, please see https://journals.plos.org/plosone/s/recommended-repositories.

If there are ethical or legal restrictions on sharing a de-identified data set, please explain them in detail (e.g., data contain potentially sensitive information, data are owned by a third-party organization, etc.) and who has imposed them (e.g., an ethics committee). Please also provide contact information for a data access committee, ethics committee, or other institutional body to which data requests may be sent. If data are owned by a third party, please indicate how others may request data access.

5. If the reviewer comments include a recommendation to cite specific previously published works, please review and evaluate these publications to determine whether they are relevant and should be cited. There is no requirement to cite these works unless the editor has indicated otherwise.

[Note: HTML markup is below. Please do not edit.]

Reviewers' comments:

Reviewer's Responses to Questions

**Comments to the Author**

1. Is the manuscript technically sound, and do the data support the conclusions?

The manuscript must describe a technically sound piece of scientific research with data that supports the conclusions. Experiments must have been conducted rigorously, with appropriate controls, replication, and sample sizes. The conclusions must be drawn appropriately based on the data presented.

Reviewer #1: Partly

Reviewer #2: Yes

Reviewer #3: Partly

**********

2. Has the statistical analysis been performed appropriately and rigorously?

Reviewer #1: Yes

Reviewer #2: Yes

Reviewer #3: Yes

**********

3. Have the authors made all data underlying the findings in their manuscript fully available?

The PLOS Data policy requires authors to make all data underlying the findings described in their manuscript fully available without restriction, with rare exception (please refer to the Data Availability Statement in the manuscript PDF file). The data should be provided as part of the manuscript or its supporting information, or deposited to a public repository. For example, in addition to summary statistics, the data points behind means, medians and variance measures should be available. If there are restrictions on publicly sharing data—e.g. participant privacy or use of data from a third party—those must be specified.

Reviewer #1: Yes

Reviewer #2: Yes

Reviewer #3: Yes

**********

4. Is the manuscript presented in an intelligible fashion and written in standard English?

PLOS ONE does not copyedit accepted manuscripts, so the language in submitted articles must be clear, correct, and unambiguous. Any typographical or grammatical errors should be corrected at revision, so please note any specific errors here.

Reviewer #1: Yes

Reviewer #2: No

Reviewer #3: No

**********

5. Review Comments to the Author

Please use the space provided to explain your answers to the questions above. You may also include additional comments for the author, including concerns about dual publication, research ethics, or publication ethics. (Please upload your review as an attachment if it exceeds 20,000 characters)

Reviewer #1: major revision

Reasons.

As the author mentions in the results section, with the heading (Genetic Diversity and Shannon Diversity Index) and table (Table 5. Percent Coefficient of variation and Shannon diversity index in Rubus fruiticosus seed protein profile using SDS-PAGE), the CV values exceed (35.36-93.54%) the general acceptable limits of research experiments.

Other Major/minor comments on the manuscript

2. In abstract, authors must highlight the statistical research design used for quantitative traits.

3. Avoid scientific names in keywords; if added, then they should be italic.

4. In the introduction, the authors used 10-27 years or older in-text citations, which should be recent ones. The author should read the manuscript carefully; some sentences require correction to make them more accurate and scientifically sound.

5. In the material and methods section

i. Enlist the studies' landraces' names, if possible, then also add origins.

ii. Also mentioned clearly, for an ethnobotanical study, any survey conducted or just literature cited data added, as mentioned in Table 1.

iii. Mention the exact date of the experiment start and end date/seasons, along with the research design.

6. In the results section, Table 6 shows the phenolic identifications of 7 landraces, whereas in the materials and methods, 15 landraces are mentioned, which are studied. Correlation analysis should be represented in a figure instead of a table. Similarly, in the PCA, no need to add the table and eigenvalue values; just add the PCA biplot. In Figure 1, there is an overlap of values.

7. In the discussion part, the current manuscript is supported by a study with CV values is 12.44, which is acceptable in research experiments, while in the present study, the CV is above the acceptable limit, indicating that the collected data is not statistically significant. Add the latest and a few other studies to support the discussion part.

8. In the conclusion section, levels of physical are mentioned. In the study, no physical traits are studied; morphological and biochemical traits are studied.

9. Need to set referencing according journal format.

Reviewer #2: Kindly refer the suggestions in attachment for the manuscript Biochemical Profiling and Ethnobotanical Uses of Rubus fruticosus L. in Khyber Pakhtunkhwa, Pakistan.

The MS needs major revision before considering it.

Reviewer #3: Please address the following points

1. The article combines many studies such as ethnobotanical documentation, morphological characterization, seed protein profiling and phytochemical analysis, however, combining all these studies are insufficiently justified. The objective of the study is quite broad and divided into many parts, which seems quite difficult to understand the main theme and hypothesis of the study. I will suggest that the authors should clearly define wether the study primarily address conservation, genetic diversity, biochemical characterization, or ethnobotanical analysis, and restructure the manuscript accordingly.

2. IBPGR descriptors, SDS-PAGE seed profiling and HPLC phytochemical analysis are well established methodologies and mostly used in diversity studies. What is the novelty or new biological insights beyond descriptive variability assessment. Is it clear in the current form that the novelty relative to existing target plant diversity studies especially from south Asia, should be clearly presented.

3. As the title show the ethnobotanical uses, but the provided section do not carefully describe field based ethnobotanical data collection, informant demographics, survey methodology or validation of traditional uses. In the current form the title of the work is misleading.

4. Who identified these plants must be mentioned in material and methods

5. The results presented are mainly descriptive, reporting ranges, percentages and variability without provide sufficient biological interpretation. Most of the statements are already presented in the tables and figures. The authors should focus on highlighting key patterns, relationships and meaningful data rather than repeating data.

6. There is no detailed information regarding the environmental condition, replication within sites, that can influence morphological traits.

7. In my places the species names are not italic, also the pattern is not uniform

8. The whole manuscript should be check for typo errors, also some of the sentences need to be organized well

**********

6. PLOS authors have the option to publish the peer review history of their article (what does this mean?). If published, this will include your full peer review and any attached files.

If you choose “no”, your identity will remain anonymous but your review may still be made public.

**Do you want your identity to be public for this peer review?**  For information about this choice, including consent withdrawal, please see our Privacy Policy.

Reviewer #1: **Yes:** Imran Ul Haq

Reviewer #2: No

Reviewer #3: No

**********

[NOTE: If reviewer comments were submitted as an attachment file, they will be attached to this email and accessible via the submission site. Please log into your account, locate the manuscript record, and check for the action link "View Attachments". If this link does not appear, there are no attachment files.]

To ensure your figures meet our technical requirements, please review our figure guidelines: https://journals.plos.org/plosone/s/figures

You may also use PLOS’s free figure tool, NAAS, to help you prepare publication quality figures: https://journals.plos.org/plosone/s/figures#loc-tools-for-figure-preparation.

NAAS will assess whether your figures meet our technical requirements by comparing each figure against our figure specifications.

Attachment Submitted filename: Comments R.docx

# Author comment: Author response to Decision Letter 1

**DOI:** 10.1371/journal.pone.0350451.r002

20 Apr 2026

We sincerely thank the Editor and Reviewer for their valuable time, constructive comments, and insightful suggestions, which have greatly improved the quality of our manuscript.

We have carefully addressed all the comments raised. A point-by-point response has been provided, and all necessary revisions have been incorporated into the revised manuscript. Changes include improvements in methodology description, results interpretation, figure and table presentation, language correction, and reference formatting.

We hope that the revised manuscript now meets the expectations and standards of the journal.

Please submit your revised manuscript by Apr 19 2026 11:59PM.

1. Please ensure that your manuscript meets PLOS ONE's style requirements, including those for file naming. The PLOS ONE style templates can be found at

https://journals.plos.org/plosone/s/file?id=wjVg/PLOSOne_formatting_sample_main_body.pdf and

https://journals.plos.org/plosone/s/file?id=ba62/PLOSOne_formatting_sample_title_authors_affiliations.pdf

Response:

Thank you for this important reminder. We confirm that the manuscript has been carefully revised to fully comply with the PLOS ONE style requirements.

Specifically, we have:

Ensured the manuscript is formatted according to the PLOS ONE template guidelines for both the main body and title page

Verified and standardized file naming conventions as per journal instructions

Checked all sections to ensure consistency with PLOS ONE formatting requirements

Ensured references, tables, figures, and supporting information follow the required Vancouver style and journal structure

We have also reviewed the manuscript using the provided PLOS ONE formatting sample documents to ensure full compliance.

2. In your Methods section, please provide additional information regarding the permits you obtained for the work. Please ensure you have included the full name of the authority that approved the field site access and, if no permits were required, a brief statement explaining why.

Response: We thank the reviewer for this suggestion. We have revised the manuscript accordingly and included the full name of the approving authority along with a brief statement explaining that” The study was conducted in compliance with applicable institutional, national, and international guidelines for field collection of plant material. Ethical approval was granted by the Institutional Ethics Committee of the University of Malakand. Collection of plant material from the study sites did not require special government permits, as the sampled areas constitute non-protected forest zones in the Malakand Division; however, all collections adhered to local land-use regulations” on pager no. 04, line no. 146 to 153.

3. Please include your tables as part of your main manuscript and remove the individual files. Please note that supplementary tables (should remain/ be uploaded) as separate "supporting information" files.

Response: We thank the reviewer for this instruction. We have revised the manuscript accordingly by incorporating all main tables directly into the body of the manuscript at their appropriate locations. Supplementary tables have been retained as separate "Supporting Information" files and uploaded accordingly. We hope these revisions meet the journal's formatting requirements.

4. We note that your Data Availability Statement is currently as follows: All relevant data are contained within the manuscript and its Supporting Information files.

Please confirm at this time whether or not your submission contains all raw data required to replicate the results of your study. Authors must share the “minimal data set” for their submission. PLOS defines the minimal data set to consist of the data required to replicate all study findings reported in the article, as well as related metadata and methods (https://journals.plos.org/plosone/s/data-availability#loc-minimal-data-set-definition).

For example, authors should submit the following data:

- The values behind the means, standard deviations and other measures reported;

- The values used to build graphs;

- The points extracted from images for analysis.

Authors do not need to submit their entire data set if only a portion of the data was used in the reported study.

If your submission does not contain these data, please either upload them as Supporting Information files or deposit them to a stable, public repository and provide us with the relevant URLs, DOIs, or accession numbers. For a list of recommended repositories, please see https://journals.plos.org/plosone/s/recommended-repositories.

If there are ethical or legal restrictions on sharing a de-identified data set, please explain them in detail (e.g., data contain potentially sensitive information, data are owned by a third-party organization, etc.) and who has imposed them (e.g., an ethics committee). Please also provide contact information for a data access committee, ethics committee, or other institutional body to which data requests may be sent. If data are owned by a third party, please indicate how others may request data access.

Response: Thank you for drawing our attention to the data availability requirement. We confirm that our submission contains all raw data necessary to replicate the study findings. The values underlying all reported means, standard deviations, and statistical outputs — including those used to construct figures and tables — are provided within the manuscript text and the accompanying Supporting Information files. No additional data were generated or used beyond what is already included. We are therefore satisfied that the current Data Availability Statement accurately reflects the status of our submission.

5. If the reviewer comments include a recommendation to cite specific previously published works, please review and evaluate these publications to determine whether they are relevant and should be cited. There is no requirement to cite these works unless the editor has indicated otherwise.

Response: We have carefully reviewed the recommended publications and evaluated their relevance to our study. Where appropriate, relevant citations have been incorporated into the revised manuscript to strengthen the context and discussion. Publications that were not directly aligned with the scope and objectives of our work have not been included.

Reviewers' comments:

Reviewer's Responses to Questions

Comments to the Author

1. Is the manuscript technically sound, and do the data support the conclusions?

The manuscript must describe a technically sound piece of scientific research with data that supports the conclusions. Experiments must have been conducted rigorously, with appropriate controls, replication, and sample sizes. The conclusions must be drawn appropriately based on the data presented.

Reviewer #1: Partly

Reviewer #2: Yes

Reviewer #3: Partly

Response:

We thank Reviewers 1 and 3 for their partial assessments and Reviewer 2 for the positive evaluation. We have carefully revisited the manuscript in light of the concerns raised and have strengthened the relevant sections to ensure that the conclusions are more clearly and rigorously grounded in the data presented.

________________________________________

2. Has the statistical analysis been performed appropriately and rigorously?

Reviewer #1: Yes

Reviewer #2: Yes

Reviewer #3: Yes

Response: We are pleased that all three reviewers considered the statistical analysis appropriate and rigorous.

3. Have the authors made all data underlying the findings in their manuscript fully available?

The PLOS Data policy requires authors to make all data underlying the findings described in their manuscript fully available without restriction, with rare exception (please refer to the Data Availability Statement in the manuscript PDF file). The data should be provided as part of the manuscript or its supporting information, or deposited to a public repository. For example, in addition to summary statistics, the data points behind means, medians and variance measures should be available. If there are restrictions on publicly sharing data—e.g. participant privacy or use of data from a third party—those must be specified.

Reviewer #1: Yes

Reviewer #2: Yes

Reviewer #3: Yes

Response: We are grateful that all reviewers confirmed the data underlying the findings are fully available. Our Data Availability Statement has been reviewed and remains accurate.

________________________________________

4. Is the manuscript presented in an intelligible fashion and written in standard English?

PLOS ONE does not copyedit accepted manuscripts, so the language in submitted articles must be clear, correct, and unambiguous. Any typographical or grammatical errors should be corrected at revision, so please note any specific errors here.

Reviewer #1: Yes

Reviewer #2: No

Reviewer #3: No

Response:

We acknowledge the concerns raised by Reviewers 2 and 3 regarding language clarity. The manuscript has been carefully re-read and revised throughout to correct typographical and grammatical errors and to improve overall clarity and readability. We are confident the revised version meets the standard expected for publication in PLOS ONE.

________________________________________

5. Review Comments to the Author

Please use the space provided to explain your answers to the questions above. You may also include additional comments for the author, including concerns about dual publication, research ethics, or publication ethics. (Please upload your review as an attachment if it exceeds 20,000 characters)

Reviewer #1: major revision

Reasons.

As the author mentions in the results section, with the heading (Genetic Diversity and Shannon Diversity Index) and table (Table 5. Percent Coefficient of variation and Shannon diversity index in Rubus fruiticosus seed protein profile using SDS-PAGE), the CV values exceed (35.36-93.54%) the general acceptable limits of research experiments.

Response: We appreciate the reviewer’s careful evaluation of our results. However, it is important to clarify that the coefficient of variation (CV) values reported in table 5 now table 3 represent biological variability among Rubus fruticosus genotypes in seed storage protein profiles, rather than variability arising from experimental or methodological error.

In studies focused on genetic diversity, germplasm characterization, and biochemical marker analysis (e.g., SDS-PAGE protein profiling), relatively high CV values are expected because the purpose of the analysis is to capture polymorphism and heterogeneity among genotypes. Unlike controlled agronomic experiments where CV is commonly used as an indicator of experimental precision, in diversity studies CV reflects the extent of phenotypic or biochemical variation present within the population.

The CV values observed in our study (35.36–93.54%) correspond to differences in presence/absence and intensity of protein bands among the analyzed accessions, which is consistent with the inherent genetic heterogeneity of Rubus fruticosus populations.

Furthermore, the Shannon Diversity Index values presented in Table 5 now table 3 corroborate the presence of considerable genetic diversity, supporting the interpretation that the observed variation is biologically meaningful and relevant to the objectives of the study.

Therefore, the reported CV values should not be interpreted as exceeding acceptable experimental limits but rather as reflecting the natural genetic diversity captured by the SDS-PAGE protein profiling approach. To avoid potential misunderstanding, we have revised the manuscript to clarify that the CV values represent genotypic variability rather than experimental imprecision.

2. In abstract, authors must highlight the statistical research design used for quantitative traits.

Response: We thank the reviewer for this suggestion. The abstract has been revised to explicitly state that the experiment was conducted using a Randomized Complete Block Design (RCBD).

3. Avoid scientific names in keywords; if added, then they should be italic.

Response: We thank the reviewer for this observation. The keywords have been revised accordingly. Scientific names that were not essential as keywords have been removed. Where a scientific name has been retained due to its relevance, it has been italicized as per standard convention.

4. In the introduction, the authors used 10-27 years or older in-text citations, which should be recent ones. The author should read the manuscript carefully; some sentences require correction to make them more accurate and scientifically sound.

Response: We thank the reviewer for this valid observation. The introduction has been thoroughly revised to replace outdated citations with more recent and relevant literature, ensuring that the references better reflect the current state of knowledge in the field. Additionally, the manuscript has been carefully re-read, and sentences that were identified as unclear or insufficiently precise have been revised to improve scientific accuracy and readability throughout.

5. In the material and methods section

i. Enlist the studies' landraces' names, if possible, then also add origins.

ii. Also mentioned clearly, for an ethnobotanical study, any survey conducted or just literature cited data added, as mentioned in Table 1.

iii. Mention the exact date of the experiment start and end date/seasons, along with the research design.

Response: The studied landraces names were represented in Figure 2 and origin were included as well. The ethnobotanical information was excluded from the study and the study date and year were mentioned as suggested.

6. In the results section, Table 6 shows the phenolic identifications of 7 landraces, whereas in the materials and methods, 15 landraces are mentioned, which are studied. Correlation analysis should be represented in a figure instead of a table. Similarly, in the PCA, no need to add the table and eigenvalue values; just add the PCA biplot. In Figure 1, there is an overlap of values.

Response: Thank you for highlighting this point. Although fifteen landraces were initially included in the study (as described in the Materials and Methods section), only seven landraces are presented in Table 6 for phenolic identification. This is because detailed phenolic profiling was conducted on a subset of representative landraces selected based on their variability and significant differences observed in preliminary analyses (e.g., total phenolic content and antioxidant activity). The remaining landraces were excluded from this specific analysis due to limited variation and/or lower relevance to the objective of detailed compound identification.

Correlation analysis was represented as Figure 3 and the PCA results table showing eigenvalues has been removed, and only the PCA biplot has been retained in the revised manuscript as figure 4.

7. In the discussion part, the current manuscript is supported by a study with CV values is 12.44, which is acceptable in research experiments, while in the present study, the CV is above the acceptable limit, indicating that the collected data is not statistically significant. Add the latest and a few other studies to support the discussion part.

Response: Thank you for this important observation. We acknowledge that the coefficient of variation (CV) reported in the present study is higher than that cited in some previous studies (e.g., CV = 12.44). However, CV values are highly dependent on the type of experiment, biological material, and environmental variability, and therefore cannot be interpreted using a single universal threshold.

8. In the conclusion section, levels of physical are mentioned. In the study, no physical traits are studied; morphological and biochemical traits are studied.

Response: Thank you for your careful observation. We agree that the term “physical traits” was inaccurately used in the conclusion section. The present study specifically focuses on morphological and biochemical traits, and no physical traits were evaluated.

Accordingly, the conclusion has been revised to replace “physical traits” with “morphological and biochemical traits” to ensure consistency and accuracy with

Attachment Submitted filename: Response_to_reviewers.docx

# Peer review: Decision Letter 1

**DOI:** 10.1371/journal.pone.0350451.r003

**Authors:** Alia Ahmed

15 May 2026

Morpho-Biochemical Diversity and Phytochemical Profiling of Rubus fruticosus L. Landraces

PONE-D-25-56551R1

Dear Dr. Khan,

We’re pleased to inform you that your manuscript has been judged scientifically suitable for publication and will be formally accepted for publication once it meets all outstanding technical requirements.

Within one week, you’ll receive an e-mail detailing the required amendments. When these have been addressed, you’ll receive a formal acceptance letter and your manuscript will be scheduled for publication.

An invoice will be generated when your article is formally accepted. Please note, if your institution has a publishing partnership with PLOS and your article meets the relevant criteria, all or part of your publication costs will be covered. Please make sure your user information is up-to-date by logging into Editorial Manager at Editorial Manager® and clicking the ‘Update My Information' link at the top of the page. For questions related to billing, please contact billing support.

If your institution or institutions have a press office, please notify them about your upcoming paper to help maximize its impact. If they’ll be preparing press materials, please inform our press team as soon as possible -- no later than 48 hours after receiving the formal acceptance. Your manuscript will remain under strict press embargo until 2 pm Eastern Time on the date of publication. For more information, please contact onepress@plos.org.

Kind regards,

Alia Ahmed

Academic Editor

PLOS One

Additional Editor Comments (optional):

Reviewers' comments:

Reviewer's Responses to Questions

**Comments to the Author**

1. If the authors have adequately addressed your comments raised in a previous round of review and you feel that this manuscript is now acceptable for publication, you may indicate that here to bypass the “Comments to the Author” section, enter your conflict of interest statement in the “Confidential to Editor” section, and submit your "Accept" recommendation.

Reviewer #3: All comments have been addressed

**********

2. Is the manuscript technically sound, and do the data support the conclusions?

The manuscript must describe a technically sound piece of scientific research with data that supports the conclusions. Experiments must have been conducted rigorously, with appropriate controls, replication, and sample sizes. The conclusions must be drawn appropriately based on the data presented.

Reviewer #3: Yes

**********

3. Has the statistical analysis been performed appropriately and rigorously?

Reviewer #3: Yes

**********

4. Have the authors made all data underlying the findings in their manuscript fully available?

The PLOS Data policy requires authors to make all data underlying the findings described in their manuscript fully available without restriction, with rare exception (please refer to the Data Availability Statement in the manuscript PDF file). The data should be provided as part of the manuscript or its supporting information, or deposited to a public repository. For example, in addition to summary statistics, the data points behind means, medians and variance measures should be available. If there are restrictions on publicly sharing data—e.g. participant privacy or use of data from a third party—those must be specified.

Reviewer #3: Yes

**********

5. Is the manuscript presented in an intelligible fashion and written in standard English?

PLOS ONE does not copyedit accepted manuscripts, so the language in submitted articles must be clear, correct, and unambiguous. Any typographical or grammatical errors should be corrected at revision, so please note any specific errors here.

Reviewer #3: Yes

**********

6. Review Comments to the Author

Please use the space provided to explain your answers to the questions above. You may also include additional comments for the author, including concerns about dual publication, research ethics, or publication ethics. (Please upload your review as an attachment if it exceeds 20,000 characters)

Reviewer #3: All comments have been addressed. The paper can be accepted.........................................................................

**********

7. PLOS authors have the option to publish the peer review history of their article (what does this mean?). If published, this will include your full peer review and any attached files.

If you choose “no”, your identity will remain anonymous but your review may still be made public.

**Do you want your identity to be public for this peer review?**  For information about this choice, including consent withdrawal, please see our Privacy Policy.

Reviewer #3: No

**********

# Editor report: Acceptance letter

**DOI:** 10.1371/journal.pone.0350451.r004

**Authors:** Alia Ahmed

PONE-D-25-56551R1

PLOS One

Dear Dr. Khan,

I'm pleased to inform you that your manuscript has been deemed suitable for publication in PLOS One. Congratulations! Your manuscript is now being handed over to our production team.

At this stage, our production department will prepare your paper for publication. This includes ensuring the following:

* All references, tables, and figures are properly cited

* All relevant supporting information is included in the manuscript submission,

* There are no issues that prevent the paper from being properly typeset

You will receive further instructions from the production team, including instructions on how to review your proof when it is ready. Please keep in mind that we are working through a large volume of accepted articles, so please give us a few days to review your paper and let you know the next and final steps.

Lastly, if your institution or institutions have a press office, please let them know about your upcoming paper now to help maximize its impact. If they'll be preparing press materials, please inform our press team within the next 48 hours. Your manuscript will remain under strict press embargo until 2 pm Eastern Time on the date of publication. For more information, please contact onepress@plos.org.

You will receive an invoice from PLOS for your publication fee after your manuscript has reached the completed accept phase. If you receive an email requesting payment before acceptance or for any other service, this may be a phishing scheme. Learn how to identify phishing emails and protect your accounts at https://explore.plos.org/phishing.

If we can help with anything else, please email us at customercare@plos.org.

Thank you for submitting your work to PLOS ONE and supporting open access.

Kind regards,

PLOS ONE Editorial Office Staff

on behalf of

Dr. Alia Ahmed

Academic Editor

PLOS One
